# Author Correction: A µ-opioid receptor superagonist analgesic with minimal adverse effects

**DOI:** 10.1038/s41586-026-10552-1

**Published:** 2026-04-24

**Authors:** Juan L. Gomez, Emilya N. Ventriglia, Zachary J. Frangos, Agnieszka Sulima, Michael J. Robertson, Michael D. Sacco, Reece C. Budinich, Ilinca M. Giosan, Tongzhen Xie, Oscar Solis, Anna E. Tischer, Jennifer M. Bossert, Kiera E. Caldwell, Hannah Bonbrest, Amelie Essmann, Zelai M. Garçon-Poca, Shinbe Choi, Michael R. Noya, Feonil Limiac, Ali Arce, Grant C. Glatfelter, Margaret Robinson, Li Chen, Angelina A. Mullarkey, Dain R. Brademan, Garrett Enten, William Dunne, César Quiroz, Ingrid Schoenborn, Chae Bin Lee, Rana Rais, Daniel P. Holt, Robert F. Dannals, Lei Shi, Ruth Hüttenhain, Sergi Ferré, Eugene Kiyatkin, Jordi Bonaventura, Yavin Shaham, Venetia Zachariou, Michael H. Baumann, Georgios Skiniotis, Kenner C. Rice, Michael Michaelides

**Affiliations:** 1 Biobehavioral Imaging and Molecular Neuropsychopharmacology Section, National Institute on Drug Abuse Intramural Research Program, Baltimore, MD USA; 2 Drug Design and Synthesis Section, Molecular Targets and Medication Discovery Branch, National Institute on Drug Abuse Intramural Research Program, Baltimore, MD USA; 3 Department of Molecular and Cellular Physiology, Stanford University School of Medicine, Stanford, CA USA; 4 Department of Structural Biology, Stanford University School of Medicine, Stanford, CA USA; 5 Department of Pharmacology, Physiology and Biophysics, Boston University Chobanian and Avedisian School of Medicine, Boston, MA USA; 6 Neurobiology of Relapse Section, National Institute on Drug Abuse Intramural Research Program, Baltimore, MD USA; 7 Departament de Patologia i Terapèutica Experimental, Institut de Neurociències, Universitat de Barcelona, L’Hospitalet de Llobregat, Spain; 8 Neuropharmacology and Pain Group, Neuroscience Program, Bellvitge Institute for Biomedical Research (IDIBELL), L’Hospitalet de Llobregat, Spain; 9 Behavioral Neuroscience Research Branch, National Institute on Drug Abuse Intramural Research Program, Baltimore, MD USA; 10 Designer Drug Research Unit, National Institute on Drug Abuse Intramural Research Program, Baltimore, MD USA; 11 Computational Chemistry and Molecular Biophysics Section, National Institute on Drug Abuse Intramural Research Program, Baltimore, MD USA; 12 Integrative Neurobiology Section, National Institute on Drug Abuse Intramural Research Program, Baltimore, MD USA; 13 Johns Hopkins Drug Discovery, Johns Hopkins School of Medicine, Baltimore, MD USA; 14 Department of Neurology, Johns Hopkins School of Medicine, Baltimore, MD USA; 15 Department of Radiology, Johns Hopkins School of Medicine, Baltimore, MD USA; 16 Department of Structural Biology, St Jude Children’s Research Hospital, Memphis, TN USA; 17 Center of Excellence for Structural Cell Biology, St Jude Children’s Research Hospital, Memphis, TN USA

**Keywords:** Small molecules, Pharmacology, Addiction, Neuropathic pain

Correction to: *Nature* 10.1038/s41586-026-10299-9 Published online 1 April 2026

In the version of this article initially published, there were typographical errors where in the Fig. 3e *y*-axis label and Fig. 5a *x*-axis label, the labels now reading “Time (sec)” appeared originally as “Time (min),” while in the first sentence of the third paragraph of the “FNZ and DFNZ are selective MOR superagonists” section, the text now reading “To gain structural insights into the determinants of MOR activation and signalling by nitazenes” appeared originally as “…MOR activation by nitazene and signalling”. Also, the colors corresponding to the DFNZ traces in Fig. 6h have been changed to match the color gradient in the Fig. 6h legend. Text and figures have been amended in the HTML and PDF versions of the article.

