# Supplementary material for: A µ-opioid receptor superagonist analgesic with minimal adverse effects
Source: Nature. 2026 Apr 1;652(8112):1393–404. doi: 10.1038/s41586-026-10299-9 (PMC13128446; doi:10.1038/s41586-026-10299-9)
Supplement: Supplementary file 1 — Supplementary Methods [file 41586_2026_10299_MOESM1_ESM.pdf]

nature portfolio

https://doi.org/10.1038/s41586-026-10299-9

Supplementary information

A μ-opioid receptor superagonist analgesic with minimal adverse effects

In the format provided by the authors and unedited

Supplementary Information

A μ opioid receptor superagonist analgesic with minimal adverse effects

Juan L. Gomez[1], Emilya N. Ventriglia[1], Zachary J. Frangos[1], Agnieszka Sulima[2], Michael J. Robertson[3,4], Michael D. Sacco[3,4], Reece C. Budinich[1], Ilinca Giosan[5], Tongzhen Xie[5], Oscar Solis[1], Anna E. Tischer[1], Jennifer M. Bossert[6], Kiera E. Caldwell[6], Hannah Bonbrest[6], Amelie Essmann[7,8], Maria Z. Garçon-Poca[7,8], Shinbe Choi[9], Michael R. Noya[9], Feonil Limiac[9], Ali Arce[9], Grant Glatfelter[10], Margaret Robinson[11], Li Chen[11], Angelina A. Mullarkey[3], Dain R. Brademan[3], Garrett Enten[12], William Dunne[1], César Quiroz[12], Ingrid Schoenborn[1], Chae Bin Lee[13,14], Rana Rais[13,14,15], Daniel P. Holt[16], Robert F. Dannals[16], Lei Shi[11], Ruth Hüttenhain[3], Sergi Ferré[12], Eugene Kiyatkin[9], Jordi Bonaventura[7,8], Yavin Shaham[6], Venetia Zachariou[5], Michael H. Baumann[10], Georgios Skiniotis[3,4,17,18*], Kenner C. Rice[2*], Michael Michaelides[1]*

[1] Biobehavioral Imaging and Molecular Neuropsychopharmacology Section, National Institute on Drug Abuse Intramural Research Program, Baltimore, MD 21224 USA
[2] Drug Design and Synthesis Section, Molecular Targets and Medication Discovery Branch, National Institute on Drug Abuse Intramural Research Program, Baltimore, MD 21224 USA
[3] Department of Molecular and Cellular Physiology, Stanford University School of Medicine, Stanford, CA, 94305 USA.
[4] Department of Structural Biology, Stanford University School of Medicine, Stanford, CA, 94305 USA.
[5] Department of Pharmacology, Physiology & Biophysics, Boston University, Boston, MA, 02118 USA
[6] Neurobiology of Relapse Section, National Institute on Drug Abuse Intramural Research Program, Baltimore, MD 21224 USA
[7] Departament de Patologia i Terapèutica Experimental, Institut de Neurociències, Universitat de Barcelona, L'Hospitalet de Llobregat, Catalonia, Spain
[8] Neuropharmacology & Pain Group, Neuroscience Program, Bellvitge Institute for Biomedical Research (IDIBELL), L'Hospitalet de Llobregat, Catalonia, Spain
[9] Behavioral Neuroscience Research Branch, National Institute on Drug Abuse Intramural Research Program, Baltimore, MD 21224 USA
[10] Designer Drug Research Unit, National Institute on Drug Abuse Intramural Research Program, Baltimore, MD 21224 USA
[11] Computational Chemistry and Molecular Biophysics Section, National Institute on Drug Abuse Intramural Research Program, Baltimore, MD 21224 USA
[12] Integrative Neurobiology Section, National Institute on Drug Abuse Intramural Research Program, Baltimore, MD 21224 USA
[13] Johns Hopkins Drug Discovery, Johns Hopkins School of Medicine, Baltimore, MD 21205 USA
[14] Department of Neurology, Johns Hopkins School of Medicine, Baltimore, MD 21205 USA
[15] Department of Pharmacology, Johns Hopkins School of Medicine, Baltimore, MD 21205 USA
[16] Department of Radiology, Johns Hopkins School of Medicine, Baltimore, MD, 21205, USA.
[17] Department of Structural Biology, St. Jude Children's Research Hospital, Memphis, TN, USA
[18] Center of Excellence for Structural Cell Biology, St. Jude Children's Research Hospital, Memphis, TN, USA

*Corresponding authors:

Georgios Skiniotis, PhD georgios.skiniotis@stjude.org
Kenner C. Rice, PhD kennerr@nida.nih.gov
Michael Michaelides. PhD mike.michaelides@nih.gov

This PDF file includes:

Supplementary Table 1 Legend 3

Supplementary Methods

- **Figure 1. FNZ Synthesis** 4
- **Figure 2. DFNZ Synthesis** 5
- **Chemical synthesis information and procedures** 6-10
- **Copies of ^{1}H and ^{13}C NMR spectra and HRMS data of compound 2 – 11** 11-31
- **References** 32

Supplementary Table 1. The table lists the spatial coefficients and all proteins quantified across the μOR-APEX samples in HEK293 cells and the results of their statistical analysis over the time course of activation with DAMGO, FNZ and DFNZ.

Supplementary Figure 1. FNZ Synthesis

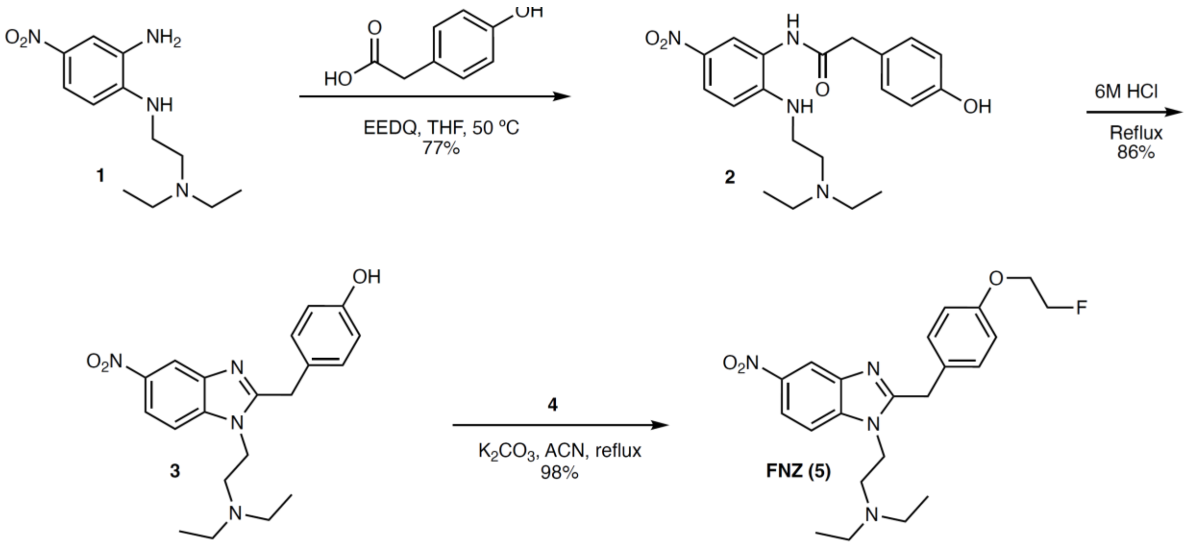

Scheme 1: Synthesis of hydroxy precursor **3** and NFZ (**5**)

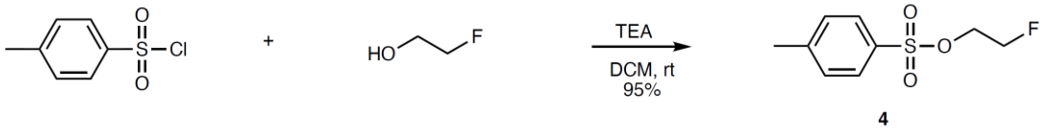

Scheme 2: Synthesis of 2-fluoroethyl tosylate (**4**)

N,*N*-Diethyl-2-(2-(4-(2-fluoroethoxy)benzyl)-5-nitro-1*H*-benzo[*d*]imidazole-1-yl)ethan-1-amine **(FNZ, 5)** and its hydroxy precursor **3** were obtained as illustrated in Scheme 1. The synthesis of compounds **3** and **5** started from an intermediate **1** that was prepared according to the reported procedure [1,2]. Condensation of **1** with phenylacetic acid in the presence of 2-ethoxy-1-ethoxycarbonyl-1,2-dihydroquinoline (EEDQ) provided the amide **2**. Subsequently, the key 2-benzylbenzimidazole intermediate **3** was obtained by HCl-induced cyclization. Alkylation of the intermediate **3** with 2-fluoroethyl tosylate (**4**)[3] yielded FNZ (**5**). The free base of **5** was converted to the hydrochloride salt upon treatment with hydrochloric acid. 2-Fluoroethyl tosylate (**4)** was synthesized by modification of the literature procedure by treatment of 2-fluoroethanol with *p*-toluenesulfonyl chloride in the presence of triethylamine (TEA) in dichloromethane (DCM)[3] (Scheme 2).

Supplementary Figure 2. DFNZ Synthesis

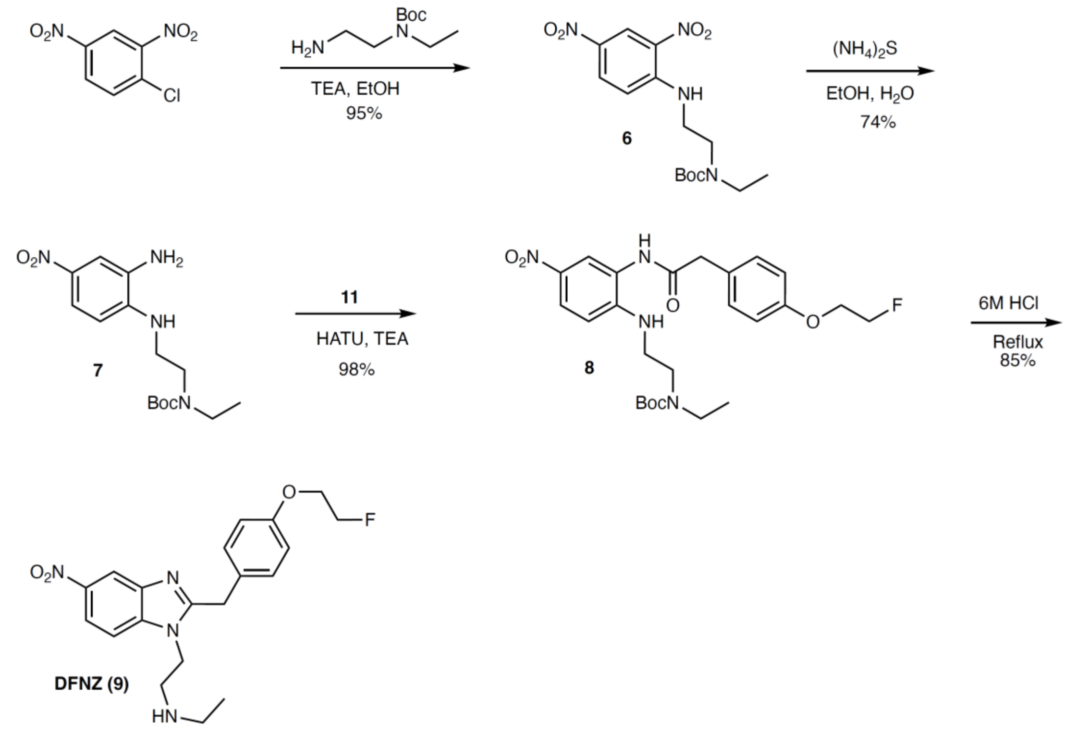

Scheme 3: Synthesis of DNFZ (**9**)

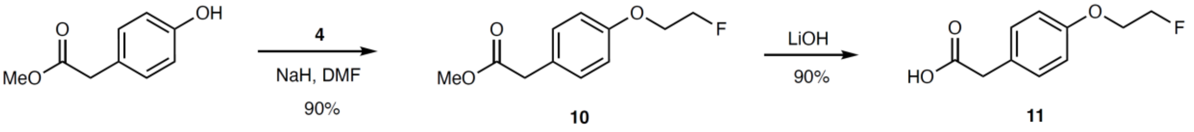

Scheme 4: Synthesis of 2-(4-(2-fluoroethoxy)phenyl)acetic acid (**11**)

Preparation of the metabolite **DFNZ** (**9**) was achieved as outlined in Scheme 3. The 1,2-diaminobenzene intermediate **7** was obtained following the reported procedure to synthesize **1** using *tert*-butyl (2-aminoethyl)(ethyl)carbamate in place of *N,N*-diethylethylenediamine. Coupling of compound **7** with 2-(4-(2-fluoroethoxy)phenyl)acetic acid (**11**) in the presence of HATU and TEA afforded amide **8** which underwent cyclization under acidic conditions at reflux to yield the final product **9**. Compound **11** was synthesized in two steps utilizing the O-alkylation of phenol and ester hydrolysis sequence as illustrated in Scheme 4.

Chemical synthesis information and procedures

Melting points were determined on a Thomas-Hoover melting-point apparatus and were uncorrected. Proton and carbon nuclear magnetic resonance (^{1}H and ^{13}C NMR) spectra were recorded on a Varian Gemini spectrometer at 400 and 101 MHz, respectively, in $CDCl_3$ or CD_3OD with the values given in ppm (TMS as internal standard) and J (Hz) assignments of ^{1}H resonance coupling. Abbreviations were used as follows: s = singlet, bs = broad singlet, d = doublet, t = triplet, q = quartet, m = multiplet. Mass spectra (HRMS) were recorded on a Waters (Milford, MA USA) Xevo-G XS QTof mass spectrometer. Ions were produced using positive ion electrospray (ESI). Thin layer chromatography (TLC) analyses were carried out on Analtech silica gel GHLF 0.25 mm plates using different solvent systems. Visualization was accomplished under UV light or by staining in an iodine chamber. Flash column chromatography was performed on a CombiFlash® NextGen 300+ PurIon™ system using RediSep Rf normal phase silica flash columns. Robertson Microlit Laboratories, Ledgewood, N.J., performed elemental analyses, and the results were within ±0.4% of the theoretical values. Chemicals and solvents were purchased from commercial suppliers and used without further purification unless otherwise stated. *N1*-(2-(diethylamino)ethyl)-4-nitrobenzene-1,2-diamine (**1**) was synthesized according to the reported procedure[1,2]and its analytical data were in agreement with the literature[2].

***N*-(2-((2-(Diethylamino)ethyl)amino)-5-nitrophenyl)-2-(4-hydroxyphenyl)acetamide (2):** A mixture of *N1*-(2-(diethylamino)ethyl)-4-nitrobenzene-1,2-diamine (**1**)[1,2] (853 mg, 3.38 mmol), 2-(4-hydroxyphenyl)acetic acid (566 mg, 3.72 mmol) and 2-ethoxy-1-ethoxycarbonyl-1,2-dihydroquinoline (EEDQ) (836 mg, 3.38 mmol) in tetrahydrofuran (THF) (4 mL) was heated at 50 °C for 26 hours under nitrogen. The reaction mixture was cooled to room temperature and the solvent removed under vacuum. The residue was treated with hexane (3x10 mL) and vigorously stirred for 10 min each time. The hexane washes were discarded, and the residue was further purified by flash chromatography using a gradient of 5% to 20% solvent B; Solvent A: $CHCl_3$; Solvent B: a mixture of $CHCl_3$/MeOH/NH_4OH (1:1:0.05). The desired product **2** was obtained as a red oil (1.11 g, 77%) with an estimated purity of ~70%, as determined by NMR analysis. Residual impurities, consisting of unreacted staring material **1**, compound **3**, and EEDQ-derived by-products, could not be readily removed at this stage and the material was therefore carried forward without further purification. Importantly, these impurities were efficiently removed in the subsequent step following crystallization, affording analytically pure compound **3**. TLC ($CHCl_3$/MeOH/NH_4OH, 95:5:1 v/v): RF = 0.24; ^{1}H NMR (400 MHz; $CDCl_3$): δ 7.93-7.91 (m, 1H), 7.86 (d, J = 2.51 Hz, 1H), 7.07 (d, J = 8.32 Hz, 2H), 6.69 (d, J = 8.32 Hz, 2H), 6.46 (d, J = 9.20 Hz, 1H), 5.37 (s, 1H), 3.59 (s, 2H), 3.16-3.14 (m, 2H), 2.66-2.63 (m, 2H), 2.56 (q, J = 7.42 Hz, 4H), 1.12 (t, J = 7.42 Hz, 6H); ^{13}C NMR (101 MHz; $CDCl_3$): δ 171.2, 156.0, 148.8, 136.6, 130.6, 125.3, 125.0, 123.6, 120.9, 116.4, 109.5, 50.6, 46.2, 42.8, 39.9, 11.2. HRMS ESI (*m/z*): $[M + H]^+$ calcd. for $C_{20}H_{27}N_4O_4$ 387.2030, found 387.2032.

4-((1-(2-(Diethylamino)ethyl)-5-nitro-1*H*-benzo[*d*]imidazol-2-yl)methyl)phenol (3): *N*-(2-((2-(diethylamino)ethyl)amino)-5-nitrophenyl)-2-(4-hydroxyphenyl)acetamide (2) (425 mg, 1.10 mmol) was taken into 18% HCl (7 mL) and stirred at 100 °C for 2 hours under nitrogen. The reaction mixture was cooled to room temperature and the pH was adjusted to 8 with 28% ammonium hydroxide solution (NH_4OH) to precipitate the product. The precipitate was filtered off, washed with cold water (7 mL) and hexane (5 mL) to give **3** (350 mg, 86%) as a yellow solid. The product was additionally purified by crystallization from ethanol to give light-yellow crystals (319 mg), m.p.: 186-187 °C. TLC ($CHCl_3$/MeOH/NH_4OH, 95:5:1 v/v): RF = 0.65. ^{1}H NMR (400 MHz; $CDCl_3$): δ 8.60 (d, J = 2.12 Hz, 1H), 8.18 (dd, J = 8.92 and 2.12 Hz, 1H), 7.69 (s, 1H), 7.35 (d, J = 8.92 Hz , 1H), 7.00 (d, J = 8.43 Hz, 2H), 6.69 (d, J = 8.32 Hz, 2H), 4.29 (s, 2H), 4.13 (t, J = 6.62 Hz, 2H), 2.60 (t, J = 6.62 Hz, 2H), 2.48 (q, J = 7.12 Hz, 4H), 0.87 (t, J = 7.12 Hz, 6H). ^{13}C NMR (101 MHz; $CDCl_3$): δ 158.1, 155.7, 143.6, 141.4, 139.3, 129.6, 126.3, 118.3, 116.1, 115.8, 109.4, 52.1, 47.5, 43.7, 33.5, 11.7. HRMS ESI (*m/z*): $[M + H]^+$ calcd for $C_{20}H_{25}N_4O_3$ 369.1927, found 369.1924. Anal. calcd. for $C_{20}H_{24}N_4O_3 \cdot 0.4\ H_2O$: C 63.95; H 6.65; N 14.91; Found: C 63.83; H 6.44; N 14.87.

2-Fluoroethyl 4-methylbenzenesulfonate (4): To a solution of 2-fluoroethan-1-ol (5.0 g, 78 mmol) in dichloromethane (DCM) (50 mL) was added *p*-toluenesulfonyl chloride (18 g, 94 mmol) followed by triethylamine (TEA) (11.8 g, 16.3 mL, 117 mmol) at room temperature under nitrogen. The reaction mixture became heterogeneous after 10-15 min and the stirring was continued for additional 16 hours. The solvent was removed under vacuum and the residue was taken into diethyl ether (50 mL) and washed with water (40 mL). The aqueous layer was additionally extracted with diethyl ether (2x40 mL). The combined organic layers were dried over sodium sulfate (Na_2SO_4), concentrated and the residue was purified by flash chromatography using a gradient of 10% to 25% ethyl acetate in hexane to yield **4** (16.2 g, 95%) as a colorless oil. TLC (EtOAc/*n*-hexane, 1:10, v/v): RF = 0.15. NMR spectra of the product **4** agreed with the literature data[3]. ^{1}H NMR (400 MHz; $CDCl_3$): δ 7.79 (m, 2H), 7.34 (m, 2H), 4.61 (m, 1H), 4.49 (m, 1H), 4.28 (m, 1H), 4.21 (m, 1H), 2.44 (s, 3H). ^{13}C NMR (101 MHz; $CDCl_3$): δ 145.1, 132.6, 129.9, 127.91, 80.4 (d, J = 173.9 Hz), 68.4 (d, J = 21.4 Hz), 21.6.

***N,N*-Diethyl-2-(2-(4-(2-fluoroethoxy)benzyl)-5-nitro-1*H*-benzo[*d*]imidazol-1-yl)ethan-1-amine (FNZ, 5):** To a mixture of 4-((1-(2-(diethylamino)ethyl)-5-nitro-1H-benzo[d]imidazol-2-yl)methyl)phenol (3) (625 mg, 1.70 mmol) and potassium carbonate (469 mg, 3.39 mmol) in acetonitrile (10 mL) was added 2-fluoroethyl 4-methylbenzenesulfonate (4) (555 mg, 2.54 mmol) at room temperature under nitrogen. The reaction mixture was refluxed for 28 hours, cooled to room temperature and the solvent removed under vacuum. The residue was taken into a mixture of water (6 mL) and $CHCl_3$ (6 mL), the layers were separated, and the aqueous layer was additionally extracted with $CHCl_3$ (2x6 mL). The combined organic layers were dried over Na_2SO_4 and

concentrated. The resulted residue, light-brown oil, was purified by flash chromatography using a gradient of 0% to 40% solvent B; Solvent A: ethyl acetate; Solvent B: 5% methanol in ethyl acetate. The desired product **5** (690 mg, 98%) was obtained as a yellow oil that solidified upon standing at room temperature. The free base (650 mg, 1.56 mmol) was converted to the hydrochloride salt by treatment of its ethanolic solution (3 mL) with 38% hydrochloric acid (0.156 mL, 1.87 mmol) at +4 °C affording colorless needles, 550 mg, **m.p.: 183-184 °C.** TLC ($CHCl_3$/MeOH/NH_4OH, 95:5:1 v/v): RF = 0.63. ^{1}H NMR (400 MHz; $CDCl_3$): δ 8.60 (d, J = 2.09 Hz, 1H), 8.18 (dd, J = 8.92 and 2.09 Hz, 1H), 7.33 (d, J = 8.90 Hz, 1H), 7.15 (d, J = 8.60 Hz, 2H), 6.69 (d, J = 8.60 Hz, 2H), 4.78 (t, J = 4.15 Hz, 1H), 4.66 (t, J = 4.15 Hz, 1H), 4.34 (s, 2H), 4.19 (t, J = 4.16 Hz, 1H), 4.12 (t, J = 4.16 Hz, 1H), 4.08 (t, J = 6.66 Hz, 2H), 2.54 (t, J = 6.66 Hz, 2H), 2.45 (q, J = 7.12 Hz, 4H), 0.86 (t, J = 7.12 Hz, 6H). ^{13}C NMR (101 MHz; $CDCl_3$): δ 157.6, 143.5, 141.9, 139.6, 129.6, 128.1, 118.2, 116.2, 115.1, 109.3, 81.8 (d, J = 170.9 Hz), 67.2 (d, J = 20.1 Hz), 52.1, 47.6, 43.8, 33.7, 11.8. HRMS ESI (*m/z*): $[M + H]^+$ calcd for $C_{22}H_{28}N_4O_3F$ 415.2145, found 415.2148. Anal. calcd. for $C_{22}H_{28}ClFN_4O_3$ • 0.83 EtOH: C 58.09; H 6.8; N 11.45; Found: C 57.97; H 6.67; N 11.47.

***tert*-Butyl (2-((2,4-dinitrophenyl)amino)ethyl)(ethyl)carbamate (6):** To a solution of 1-chloro-2,4-dinitrobenzene (203 mg, 1.00 mmol) and triethylamine (0.181 mL, 1.3 mmol) in ethanol (3 mL) was added *tert*-butyl (2-aminoethyl)(ethyl)carbamate (226 mg, 1.20 mmol). The reaction mixture was refluxed under nitrogen for 5 hours and concentrated under reduced pressure. The residue was dissolved in ether, transferred to a separatory funnel and treated with diluted NH_4OH (pH~9) (6 mL). The layers were separated, and the aqueous layer was additionally extracted with ether (3x6 mL). The combined organic layers were dried over Na_2SO_4 and concentrated to give a yellow oil that was purified by chromatography eluting with EtOAc/Hex (15% to 80%). The desired product **6** (337 mg, 95%) was isolated as a yellow oil. TLC (Hex/EtOAc, 5:1 v/v): RF = 0.13. ^{1}H NMR (400 MHz; $CDCl_3$): δ 9.12 (s, 1H), 8.82 (bs, 1H), 8.26 (d, J = 9.47 Hz, 1H), 7.03 (bd, J = 8.19 Hz, 1H), 3.55 (s, 4H), 3.25 (bs, 2H), 1.47 (s, 9H), 1.11 (t, J = 7.0 Hz, 3H). ^{13}C NMR (101 MHz; $CDCl_3$): δ 156.1, 148.4, 136.2, 130.6, 130.3, 124.3, 113.9, 80.4, 45.4, 43.1, 42.4, 28.4, 13.8. HRMS ESI (*m/z*): $[M + Na]^+$ calcd for $C_{15}H_{22}N_4O_6Na$ 377.1437, found 377.1442.

***tert*-Butyl (2-((2-amino-4-nitrophenyl)amino)ethyl)(ethyl)carbamate (7):** In a 100 mL two-neck round bottom flask, to a solution of *tert*-butyl (2-((2,4-dinitrophenyl)amino)ethyl)(ethyl)carbamate (**6**) (708.7 mg, 2 mmol) in EtOH (7.4 mL) was added dropwise over a period of 30 min a mixture of ammonium sulfide (1.060 g, 45% Wt, 7 mmol), water (7.9 mL) and ethanol (15.8 mL) under nitrogen at 65°C. The reaction mixture was then heated at 70°C for 24 hours, cooled to room temperature and concentrated under reduced pressure to ~1/3 of the original volume. The residue was made basic (pH ~10) with NH_4OH and extracted with $CHCl_3$ (4x6 mL). The organic layers were dried over Na_2SO_4 and concentrated to give a dark-brown oil that was purified by

chromatography eluting with EtOAc/Hex (5% to 40%). The desired product **7** (477 mg, 74%) was isolated as a red oil. TLC (DCM/EtOAc, 3:1 v/v): RF = 0.68. ^{1}H NMR (400 MHz; $CDCl_3$): δ 7.72 (d, J = 8.59 Hz, 1H), 7.52 (bs, 1H), 6.38 (d, J = 7.52 Hz, 1H), 5.56 (bs, 1H), 3.31-3.20 (m, 8H), 1.43 (s, 9H), 1.08 (t, J = 7.06 Hz, 3H). ^{13}C NMR (101 MHz; $CDCl_3$): δ 157.3, 143.9, 137.8, 131.9, 118.7, 110.8, 106.9, 80.3, 45.8, 44.1, 42.9, 28.4, 13.7. HRMS ESI (*m/z*): $[M + H]^+$ calcd for $C_{15}H_{25}N_4O_4$ 325.1876, found 325.1871.

***tert*-Butyl ethyl(2-((2-(2-(4-(2-fluoroethoxy)phenyl)acetamido)-4-nitrophenyl)amino)ethyl)carbamate (8):** To a solution of *tert*-butyl (2-((2-amino-4-nitrophenyl)amino)ethyl)(ethyl)carbamate (**7**) (246 mg, 758 μmol) in DCM (5 mL) was added 2-(4-(2-fluoroethoxy)phenyl)acetic acid (**11**) (150 mg, 758 μmol) followed by HATU (303 mg, 796 μmol) and TEA (264 μL, 1.90 mmol) under nitrogen at room temperature. The reaction mixture was stirred for 1.5 hours and quenched with satd. $NaHCO_3$. The layers were separated, and the aq. layer was additionally extracted with DCM (3x10 mL). The combined organic layers were dried over Na_2SO_4 and concentrated under reduced pressure. The crude material was purified by chromatography eluting with EtOAc/DCM (2% to 20%). The desired product was isolated as a yellow oil (376 mg, 98%). TLC (DCM/EtOAc, 3:1 v/v): RF = 0.65. ^{1}H NMR (400 MHz; $CDCl_3$): δ 8.02 (bs, 1H), 7.96 (d, J = 9.10, 1H), 7.33 (d, J = 5.93 Hz, 2H), 6.99 (bs, 1H), 6.92 (d, J = 8.27 Hz, 2H), 6.46 (d, J = 7.93 Hz, 1H), 6.07 (bs, 1H), 4.79 (t, J = 3.92 Hz, 1H), 4.67 (t, J = 3.92 Hz, 1H), 4.22 (t, J = 3.92 Hz, 1H), 4.16 (t, J = 3.92 Hz, 1H), 3.73 (bs, 2H), 3.47 (bs, 2H), 3.26 (bs, 2H), 3.20 (q, J = 7.16 Hz, 2H), 1.47 (s, 9H), 1.09 (t, J = 6.78 Hz, 3H). ^{13}C NMR (101 MHz; $CDCl_3$): δ 170.8, 157.8, 157.6, 148.6, 136.8, 130.7, 127.1, 124.6, 122.9, 120.9, 115.2, 108.6, 81.8 (d, J = 171.7 Hz), 80.2, 67.1 (d, J = 30.3 Hz), 46.2, 44.2, 43.4, 42.9, 28.4, 13.6. HRMS ESI (*m/z*): $[M + H]^+$ calcd for $C_{25}H_{34}N_4O_6F$ 505.2462, found 505.2461.

N-Ethyl-2-(2-(4-(2-fluoroethoxy)benzyl)-5-nitro-1H-benzo[d]imidazol-1-yl)ethan-1-amine (9): A mixture of *tert*-butyl ethyl(2-((2-(2-(4-(2-fluoroethoxy)phenyl)acetamido)-4-nitrophenyl)amino)ethyl)carbamate (**8**) (376 mg, 0.745 mmol) and 6M HCl (5 mL) was refluxed under nitrogen for 7 hours. The reaction mixture was cooled in an ice-water bath and made basic with solid NaOH. The aq. layer was extracted with a mixture of $CHCl_3$/MeOH (4:1) (3x10 mL). The combined organic layers were dried over Na_2SO_4 and concentrated under reduced pressure. The crude material was purified by chromatography using a gradient of 15% to 60% solvent B; Solvent A: $CHCl_3$; Solvent B: a mixture of $CHCl_3$/MeOH/NH_4OH (95:5:1) to afford the product **9** (245 mg, 85%) as a light-yellow foam. The free base (210 mg, 0.546 mmol) was converted to the hydrochloride salt by treating its ethanolic solution (3 mL) with a 2N solution of HCl in ether (0.32 mL, 0.65 mmol) affording an off-white solid (207 mg), **m.p.: 211-213 °C.** TLC ($CHCl_3$/MeOH/NH_4OH, 95:5:1 v/v): RF = 0.35. ^{1}H NMR (400 MHz; $CDCl_3$): δ 8.62 (s, 1H), 8.17-8.15 (m, 1H), 7.37 (d, J = 8.90 Hz, 1H), 7.17 (d, J = 8.38 Hz, 2H), 6.86 (d, J = 8.36 Hz, 2H), 4.77 (t, J = 3.89 Hz, 1H), 4.65 (t, J = 3.89 Hz, 1H), 4.32 (s, 2H), 4.19 (t, J = 3.89 Hz, 1H),

4.18-4.11 (m, 3H), 2.77 (t, *J* = 6.51 Hz, 2H), 2.53 (q, *J* = 7.09 Hz, 2H), 0.99 (t, *J* = 7.07 Hz, 3H). ^{13}C NMR (101 MHz; $CD_3OD/CDCl_3$): *δ* 159.2, 158.9, 145.1, 142.2, 140.1, 130.8, 128.4, 119.8, 116.2, 116.0, 111.0, 82.8 (d, *J* = 170.5 Hz), 68.4 (d, *J* = 19.9 Hz), 45.7, 44.3, 41.0, 33.6, 11.4. HRMS ESI (*m/z*): $[M + H]^+$ calcd for $C_{20}H_{24}N_4O_3F$ 387.1832, found 387.1837. Anal. calcd. for $C_{20}H_{24}ClFN_4O_3$ • 1.5 H_2O: C 53.39; H 6.05; N 12.45; Found: C 53.52; H 6.02; N 12.18.

Methyl 2-(4-(2-fluoroethoxy)phenyl)acetate (10): To a suspension of NaH (238 mg, 60% Wt, 5.96 mmol) in DMF (3 mL) was added a solution of methyl 2-(4-hydroxyphenyl)acetate (900 mg, 5.42 mmol) in DMF (5 mL) dropwise at room temperature under nitrogen. The mixture was heated to 50°C and the stirring was continued for additional 30 min before a solution of 2-fluoroethyl 4-methylbenzenesulfonate (**4**) (1.30 g, 5.96 mmol) in DMF (5 mL) was added dropwise. The reaction mixture was then heated to 80°C and stirred at that temperature for 1 hour. The reaction mixture was cooled in an ice-water bath and quenched with water (10 mL). The aqueous layer was extracted with ether (4x15 mL). The combined organic layers were dried over Na_2SO_4 and concentrated under reduced pressure to afford **10** (1.04 g, 90%) as a colorless oil. The crude product (>95% pure by NMR) was used in the next step of the synthesis. ^{1}H NMR (400 MHz; $CDCl_3$): *δ* 7.19 (d, *J* = 8.46 Hz, 2H), 6.88 (d, *J* = 8.44 Hz, 2H), 4.79 (t, *J* = 4.12 Hz, 1H), 4.67 (t, *J* = 4.12 Hz, 1H), 4.22 (t, *J* = 4.12 Hz, 1H), 4.15 (t, *J* = 4.12 Hz, 1H), 3.67 (s, 3H), 3.56 (s, 2H). ^{13}C NMR (101 MHz; $CDCl_3$): *δ* 172.2, 157.5, 130.3, 126.7, 114.7, 81.98 (d, *J* = 171.6 Hz), 67.1 (d, *J* = 20.7 Hz), 51.9, 40.2.

2-(4-(2-Fluoroethoxy)phenyl)acetic acid (11): To a solution of methyl 2-(4-(2-fluoroethoxy)phenyl)acetate (**10**) (480 mg, 2.26 mmol) in THF (5 mL) was added a solution of LiOH (65.0 mg, 2.71 mmol) in water (3 mL) at room temperature under nitrogen. The reaction mixture was stirred for 3 hours, and the organic solvent was evaporated under reduced pressure. The residue was diluted with H_2O (5 mL) and extracted with ether (2x10 mL); the ether layers were discarded. The aqueous layer was acidified with 3N HCl (pH~1-2) and extracted with a mixture of $CHCl_3$/MeOH (5:1) (3x10 mL). The combined organic layers were dried over Na_2SO_4 and concentrated to give the desired product **11** (403 mg, 90%) as a white solid. The crude product (>95% pure by NMR) was used in the next step of the synthesis. ^{1}H NMR (400 MHz; CD_3OD): *δ* 7.18 (d, *J* = 8.39 Hz, 2H), 6.87 (d, *J* = 8.40 Hz, 2H), 4.74 (t, *J* = 3.86 Hz, 1H), 4.62 (t, *J* = 3.86 Hz, 1H), 4.21 (t, *J* = 3.86 Hz, 1H), 4.13 (t, *J* = 3.86 Hz, 1H), 3.51 (s, 2H). ^{13}C NMR (101 MHz; CD_3OD): *δ* 174.4, 157.6, 130.0, 127.1, 114.1, 81.73 (d, *J* = 169.9 Hz), 67.1 (d, *J* = 19.9 Hz), 39.6.

^{1}H and ^{13}C NMR spectra of compound **2**:

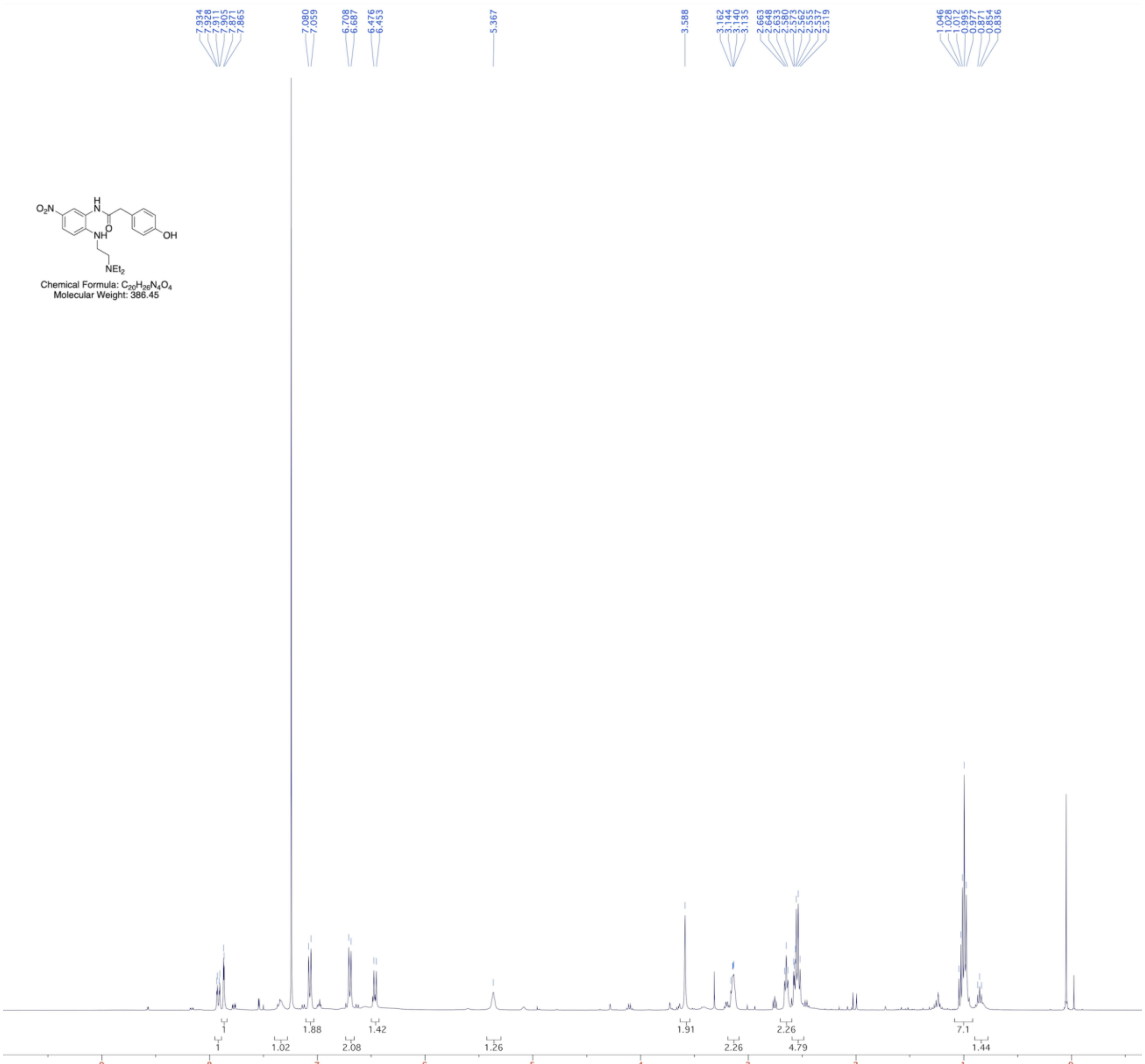

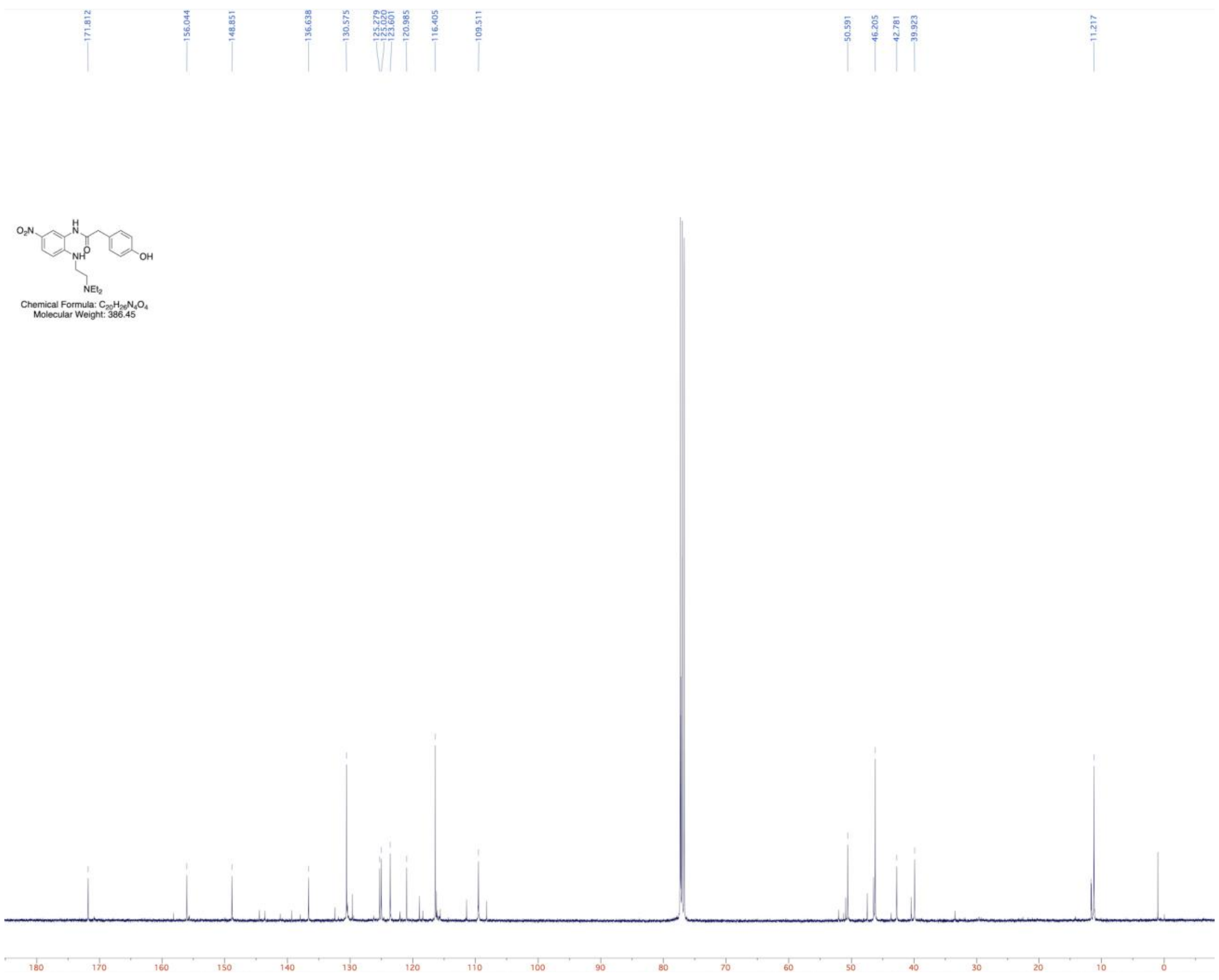
171.812
156.044
148.851
136.638
130.575
125.279
125.020
123.601
120.985
116.405
109.511
50.591
46.205
42.781
39.923
11.217
O2N
NH
NEt2
OH
Chemical Formula: C20H26N4O4
Molecular Weight: 386.45
180
170
160
150
140
130
120
110
100
90
80
70
60
50
40
30
20
10
0

HRMS data of compound **2**:

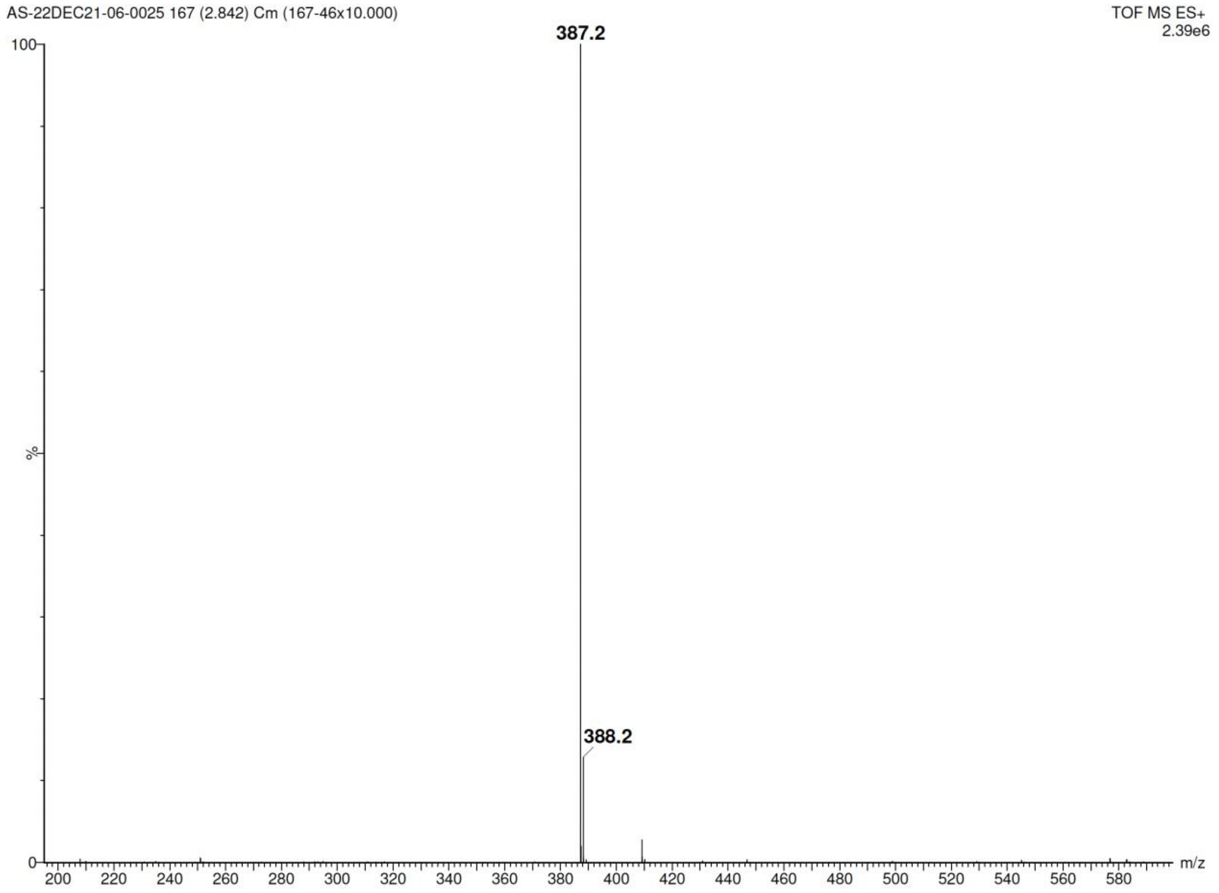

Elemental Composition Report

Page 1

Single Mass Analysis

Tolerance = 5.0 mDa / DBE: min = -1.5, max = 100.0
Element prediction: Off
Number of isotope peaks used for i-FIT = 3

Monoisotopic Mass, Even Electron Ions
72 formula(e) evaluated with 1 results within limits (up to 50 closest results for each mass)
Elements Used:
C: 0-100 H: 0-200 N: 4-4 O: 0-50
AS-22DEC21-06-0025 164 (2.791) AM2 (Ar,25000.0,0.00,0.00); ABS
TOF MS ES+

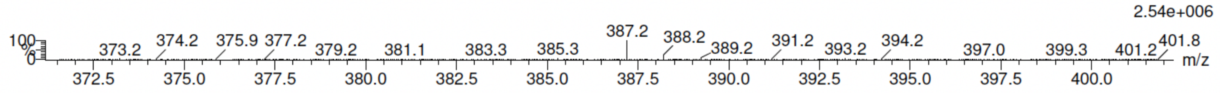

Minimum:				-1.5				
Maximum:		5.0	3.0	100.0				
Mass	Calc. Mass	mDa	PPM	DBE	i-FIT	Norm	Conf(%)	Formula
387.2030	387.2032	-0.2	-0.5	9.5	485.2	n/a	n/a	C20 H27 N4 O4

^1H and ^{13}C NMR spectra of compound **3**:

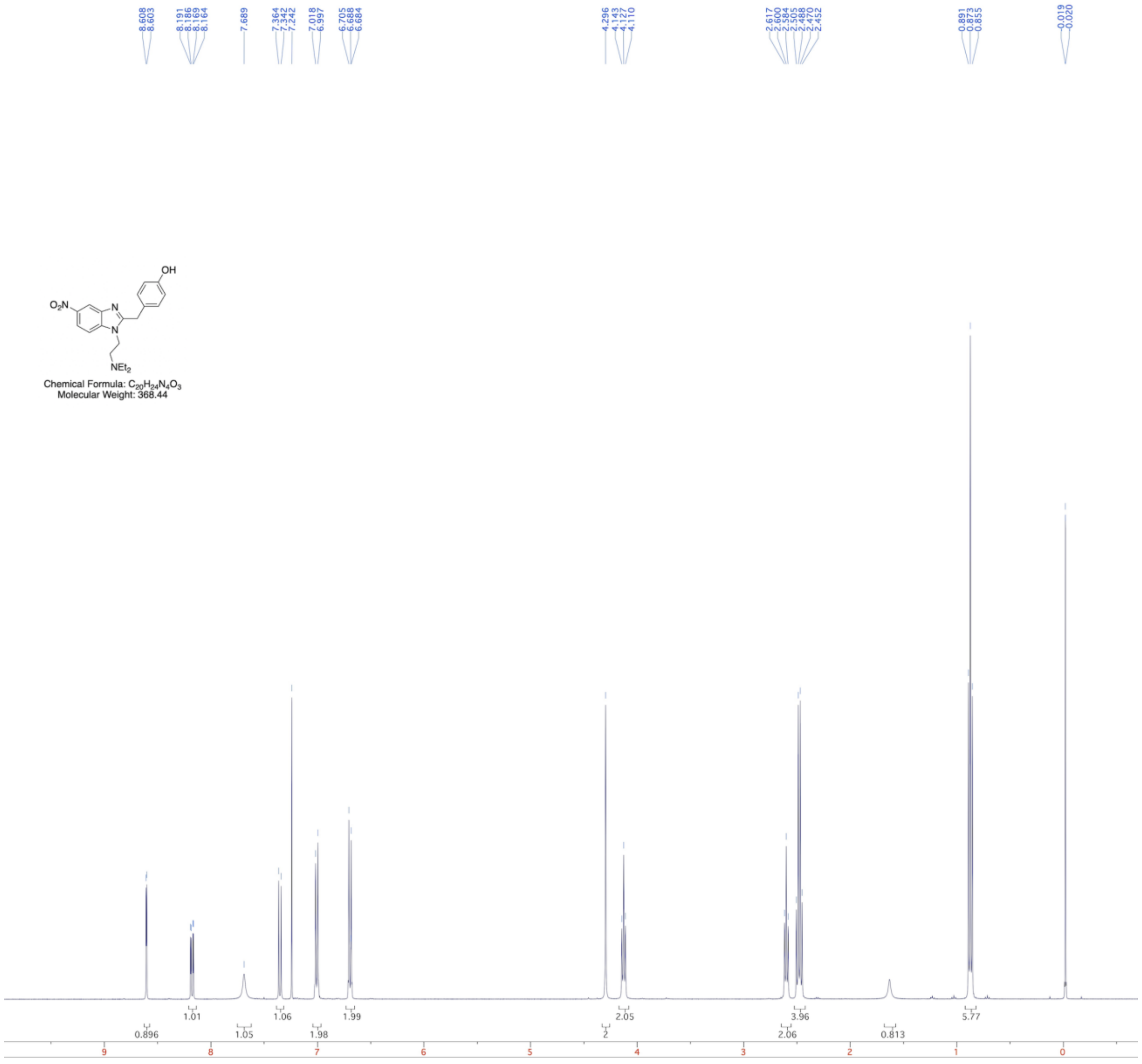

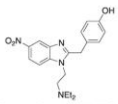

Chemical Formula: $C_{20}H_{24}N_4O_3$
Molecular Weight: 368.44

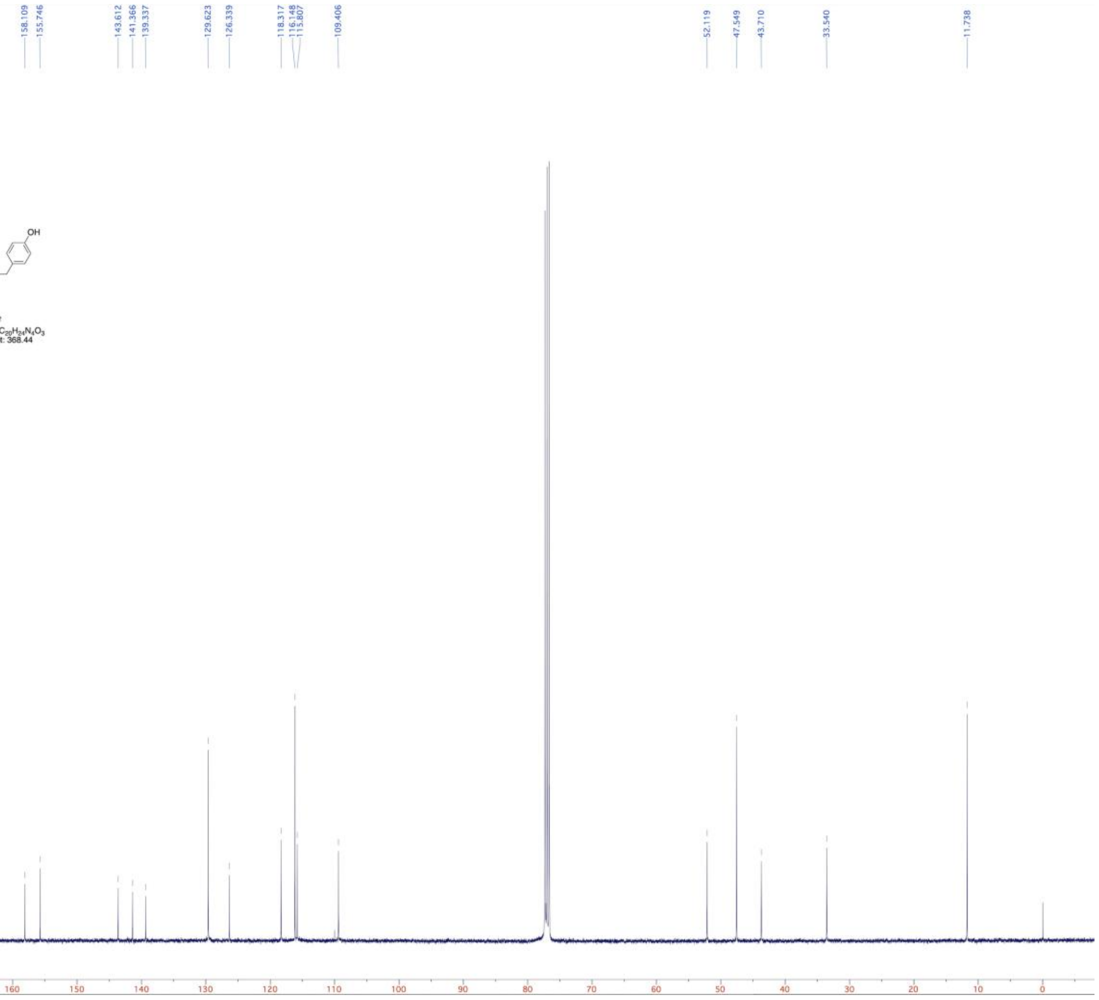

HRMS data of compound **3**:

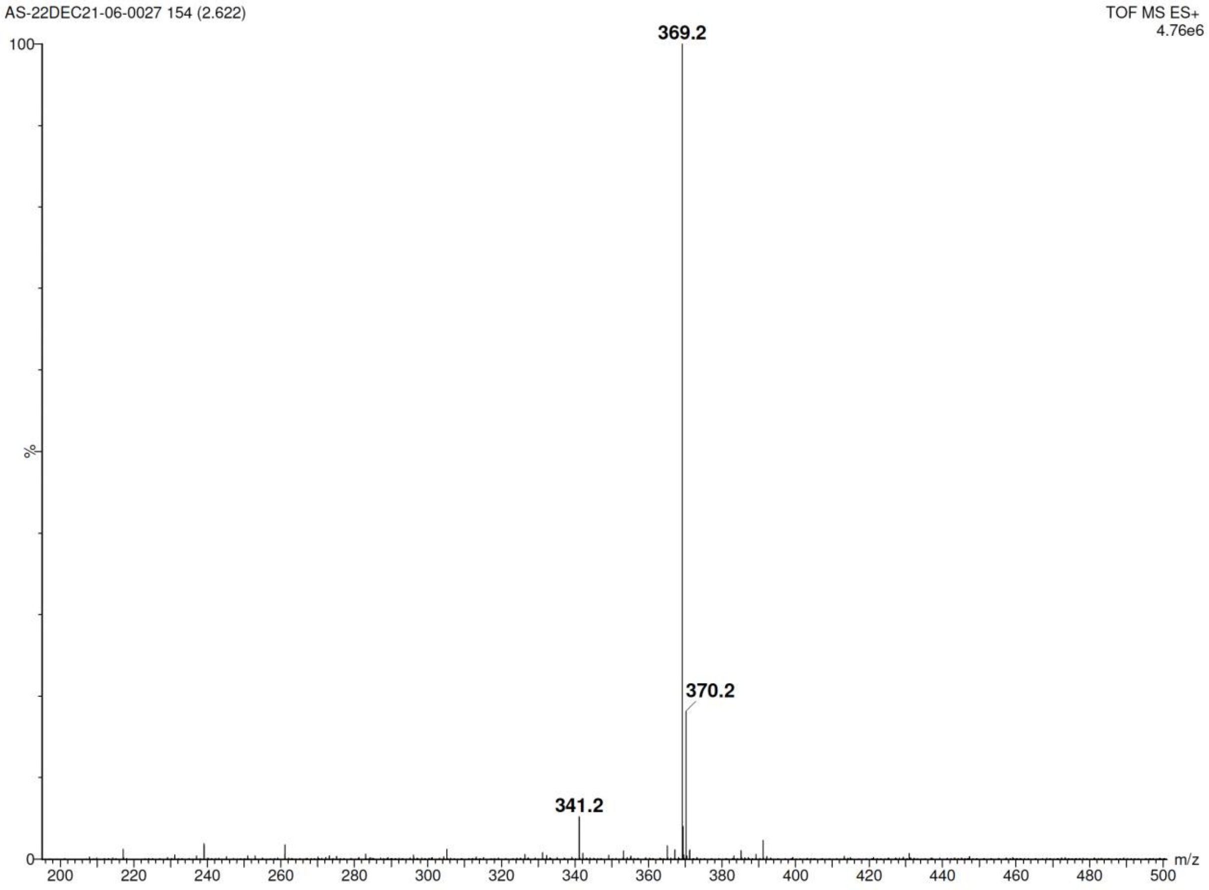

Elemental Composition Report **Page 1**

Single Mass Analysis
Tolerance = 5.0 mDa / DBE: min = -1.5, max = 100.0
Element prediction: Off
Number of isotope peaks used for i-FIT = 3

Monoisotopic Mass, Even Electron Ions
63 formula(e) evaluated with 1 results within limits (up to 50 closest results for each mass)
Elements Used:
C: 0-100 H: 0-200 N: 4-4 O: 0-50
AS-22DEC21-06-0027 151 (2.571) AM2 (Ar,25000.0,0.00,0.00); ABS
TOF MS ES+

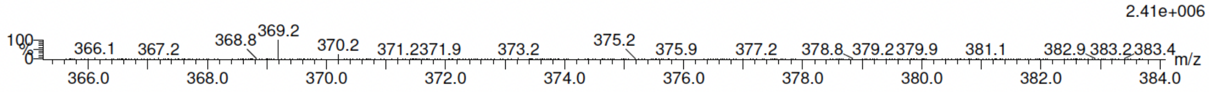

Minimum:				-1.5				
Maximum:		5.0	3.0	100.0				
Mass	Calc. Mass	mDa	PPM	DBE	i-FIT	Norm	Conf(%)	Formula
369.1924	369.1927	-0.3	-0.8	10.5	535.3	n/a	n/a	C20 H25 N4 O3

^{1}H and ^{13}C NMR spectra of compound **4**:

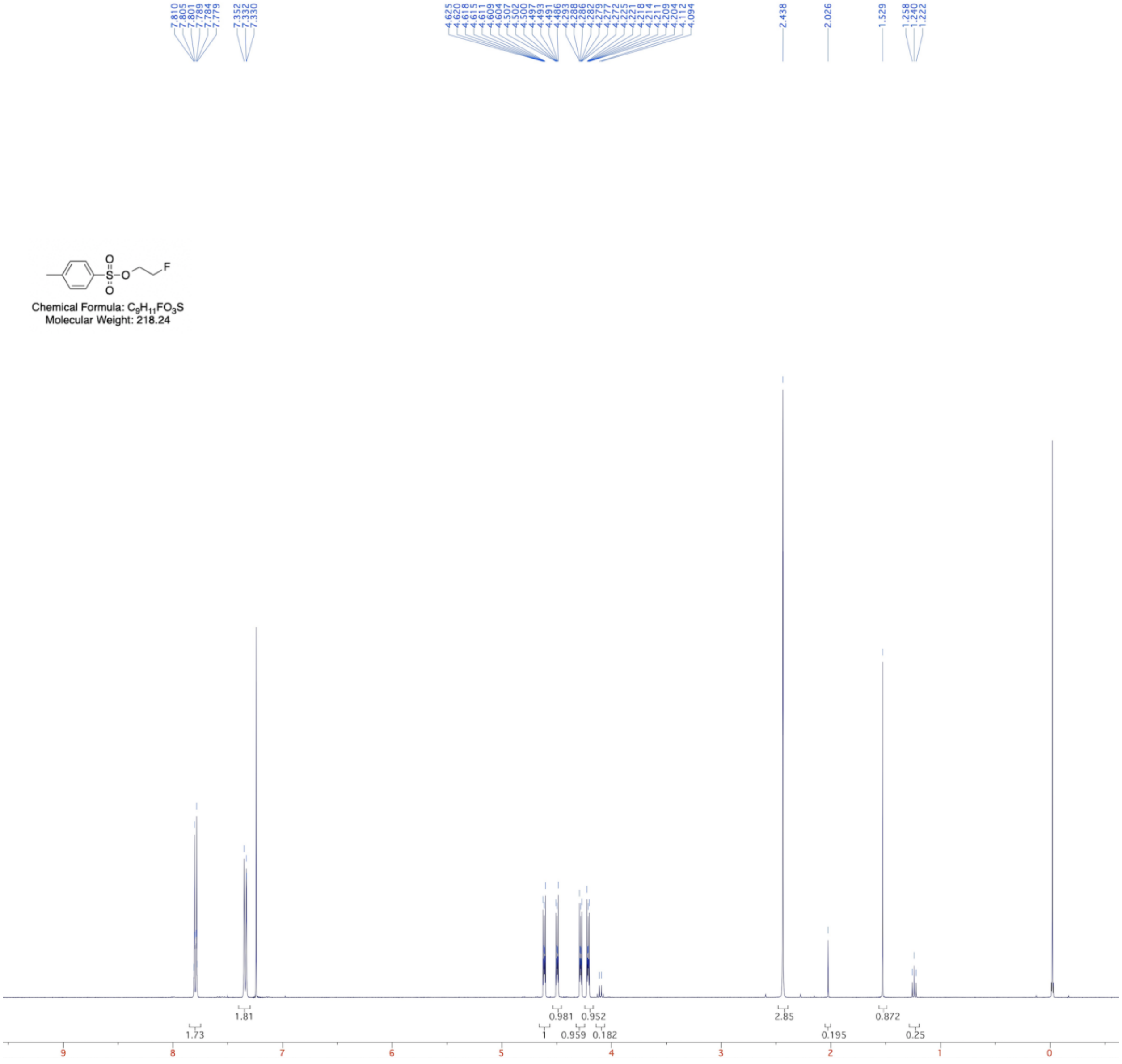

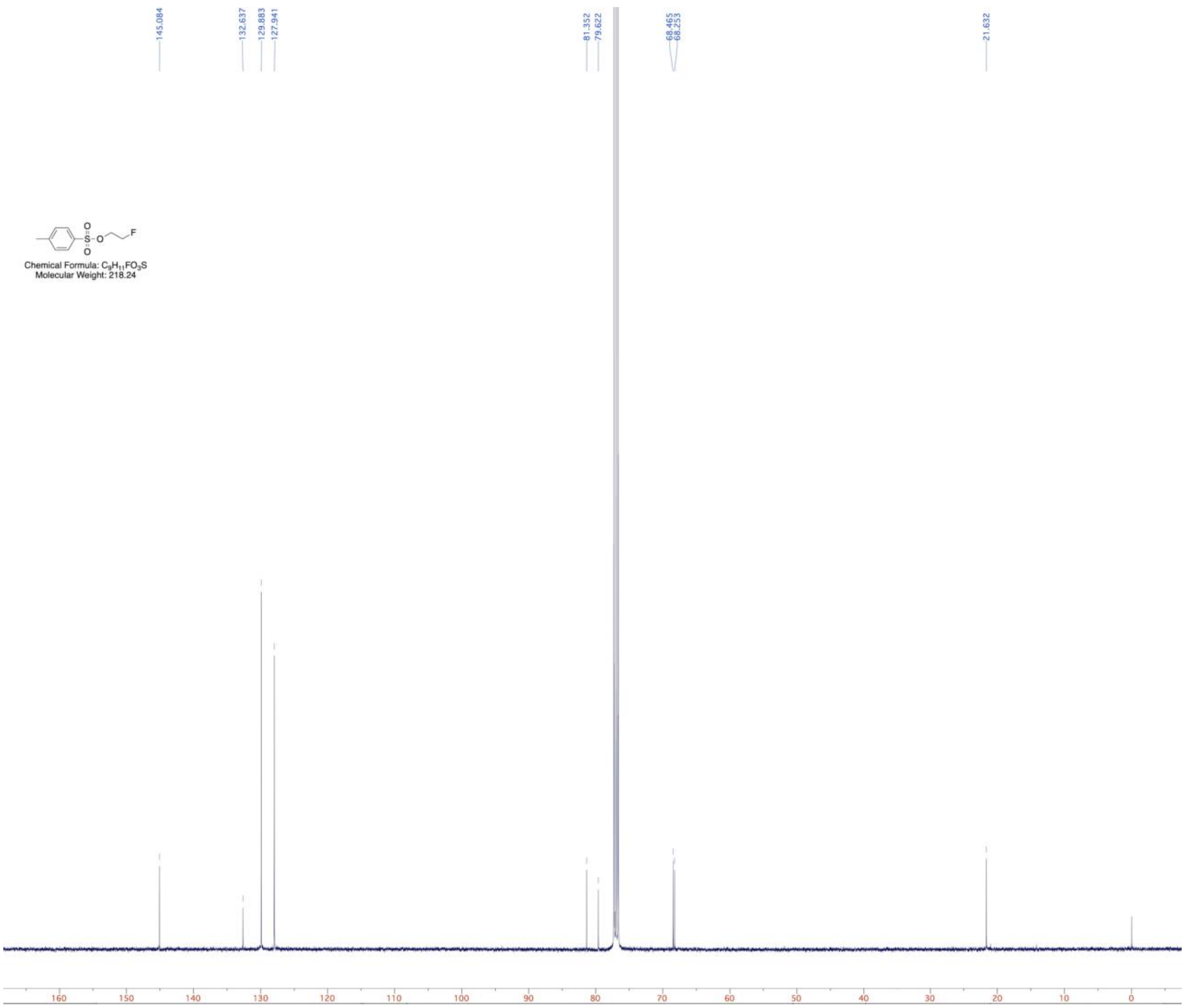
145.084
132.637
129.883
127.941
81.352
79.622
68.465
68.253
21.632
O
S
O
O
F
Chemical Formula: $C_9H_{11}FO_3S$
Molecular Weight: 218.24
160
150
140
130
120
110
100
90
80
70
60
50
40
30
20
10
0

^{1}H and ^{13}C NMR spectra of compound **5**:

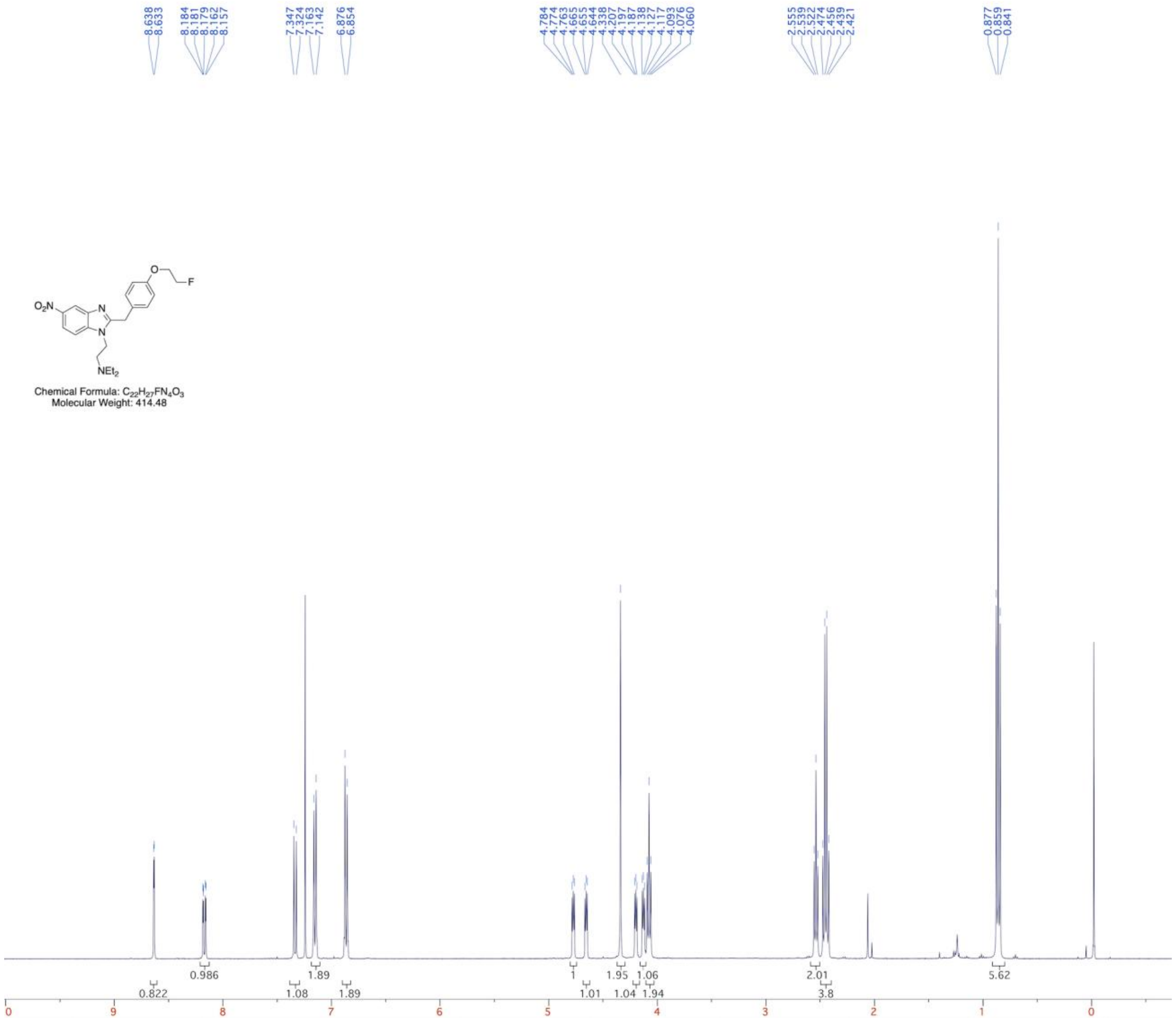

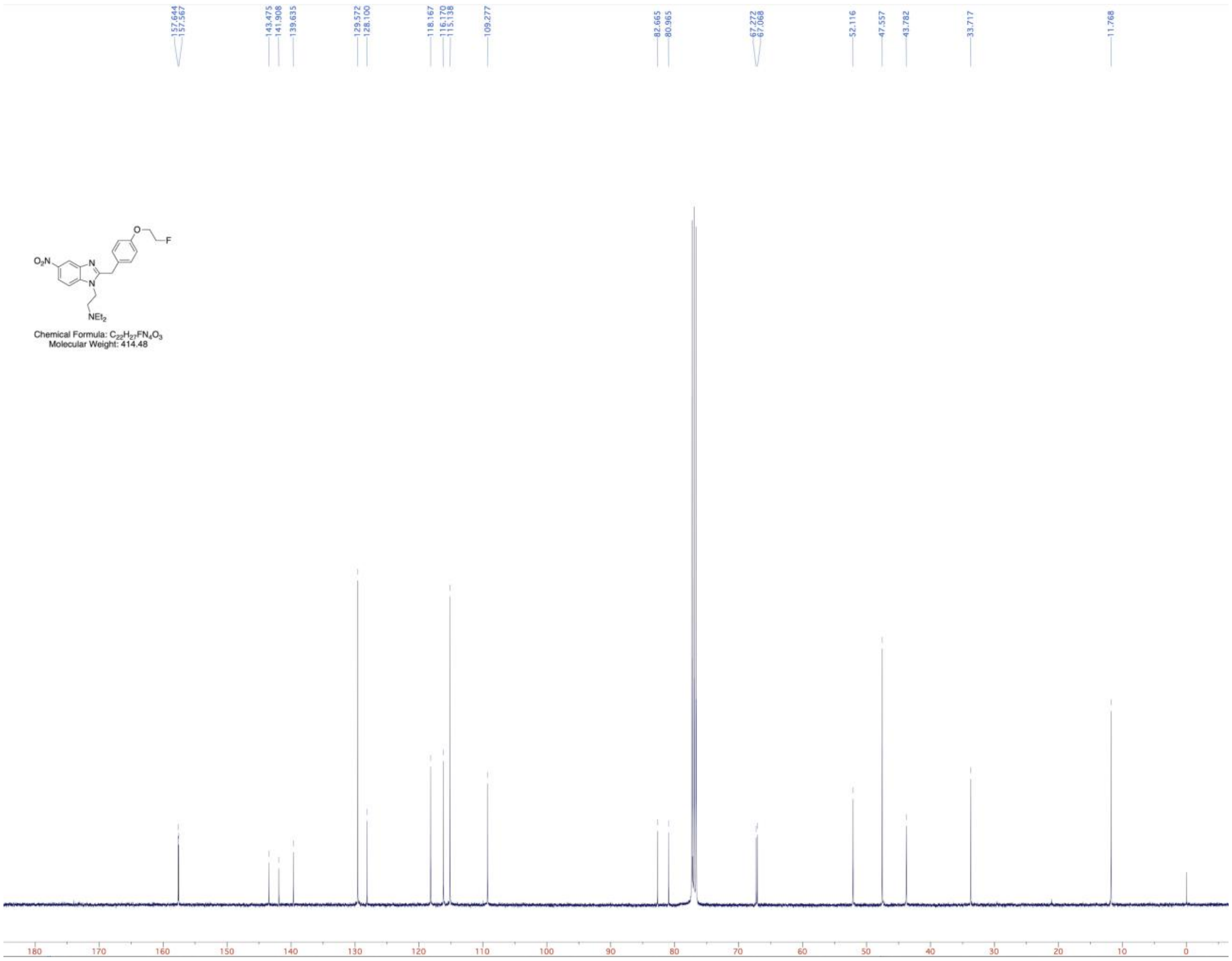

Chemical Formula: $C_{22}H_{27}FN_4O_3$
Molecular Weight: 414.48

HRMS data of compound **5**:

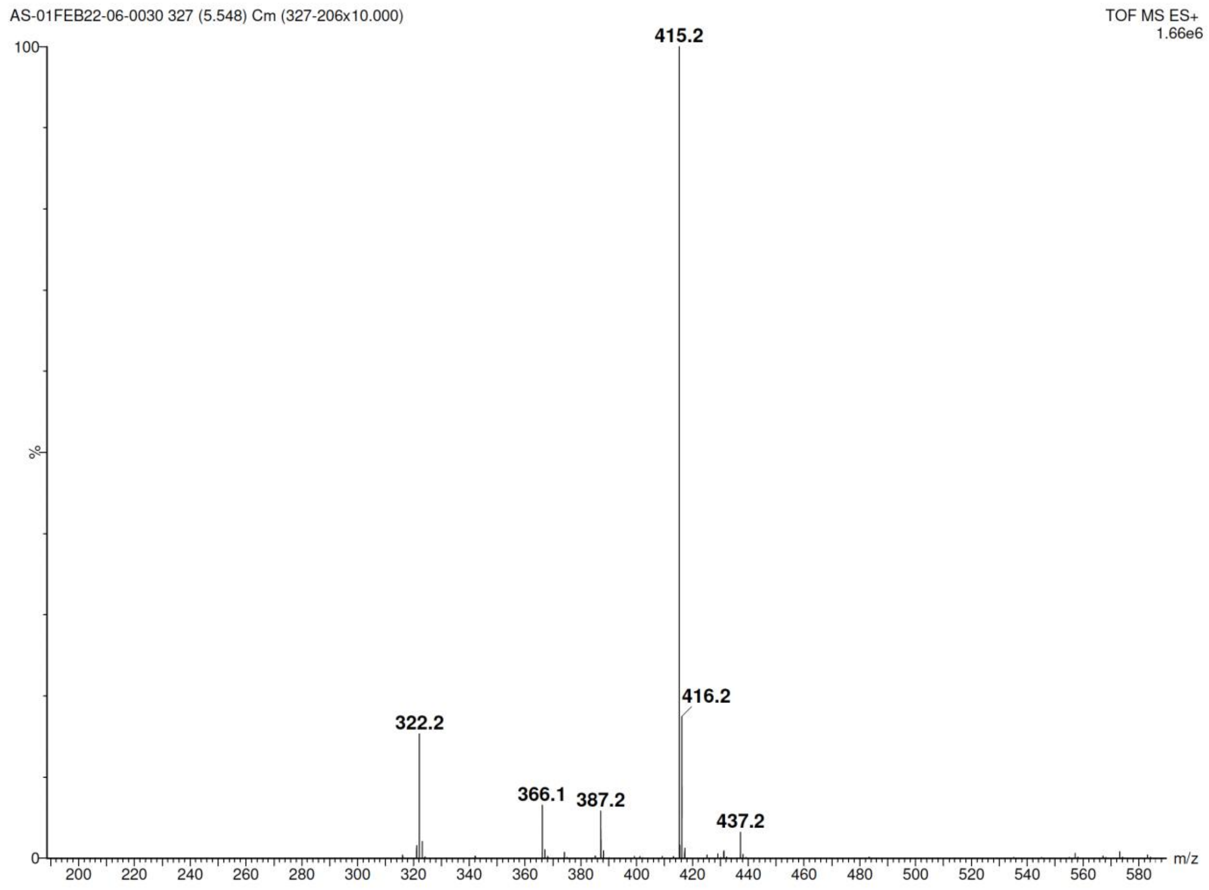

Elemental Composition Report

Page 1

Single Mass Analysis

Tolerance = 5.0 mDa / DBE: min = -1.5, max = 100.0
Element prediction: Off
Number of isotope peaks used for i-FIT = 3

Monoisotopic Mass, Even Electron Ions
72 formula(e) evaluated with 1 results within limits (up to 50 closest results for each mass)
Elements Used:
C: 0-100 H: 0-200 N: 4-4 O: 0-50 F: 1-1
AS-01FEB22-06-0030 325 (5.514) AM2 (Ar,25000.0,0.00,0.00); ABS
TOF MS ES+

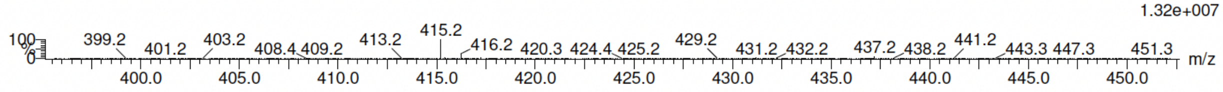

Minimum:				-1.5				
Maximum:		5.0	3.0	100.0				
Mass	Calc. Mass	mDa	PPM	DBE	i-FIT	Norm	Conf(%)	Formula
415.2148	415.2145	0.3	0.7	10.5	530.8	n/a	n/a	C22 H28 N4 O3 F

^{1}H and ^{13}C NMR spectra of compound **6**:

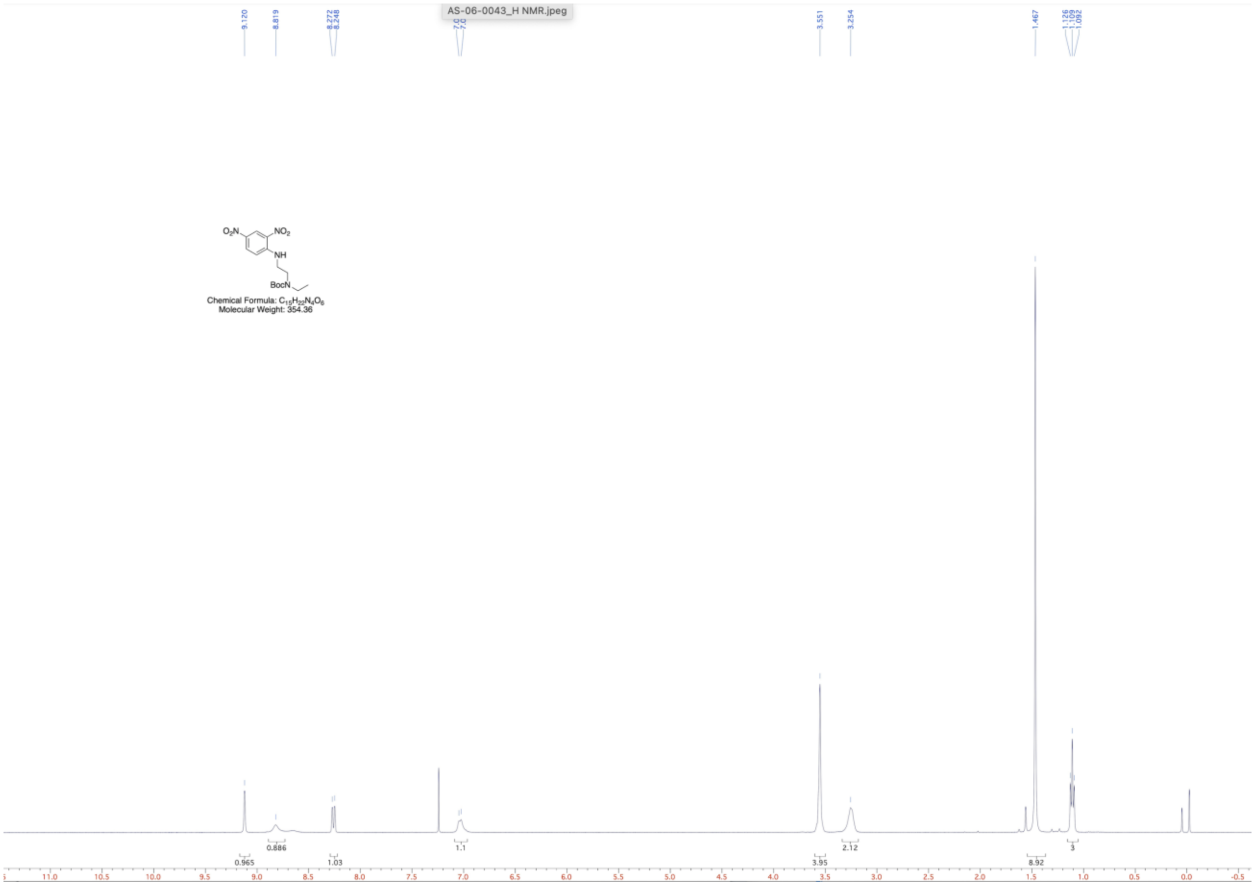

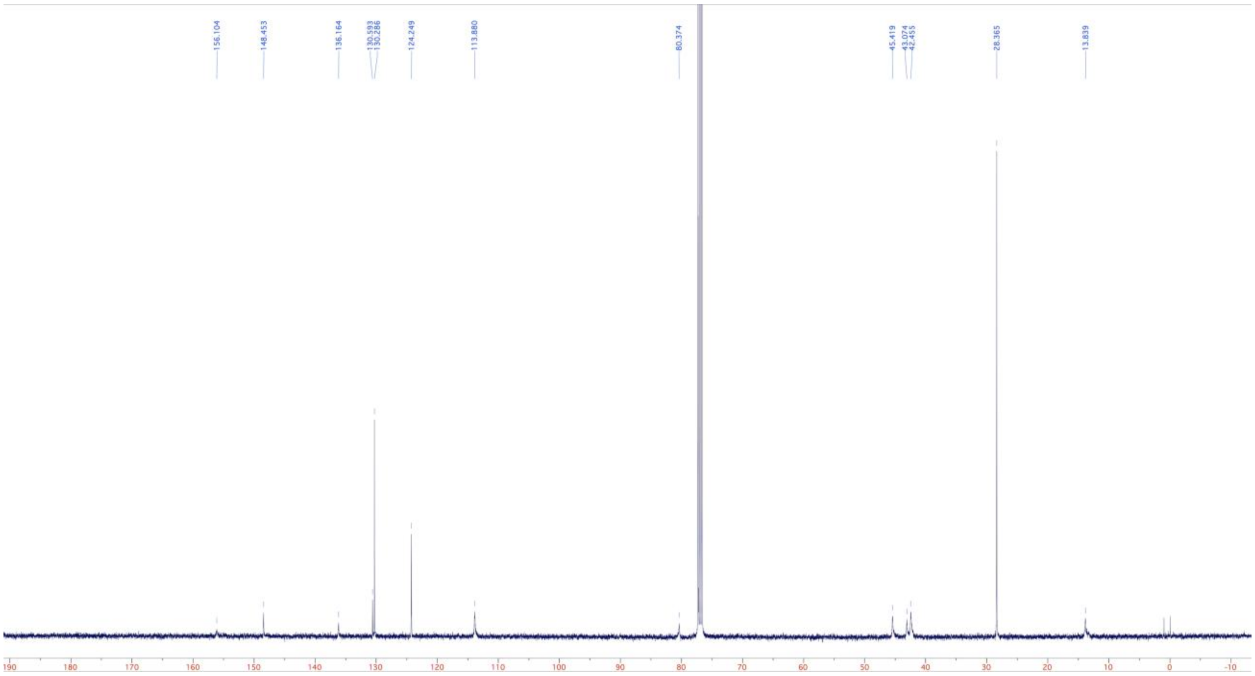

HRMS data of compound **6**:

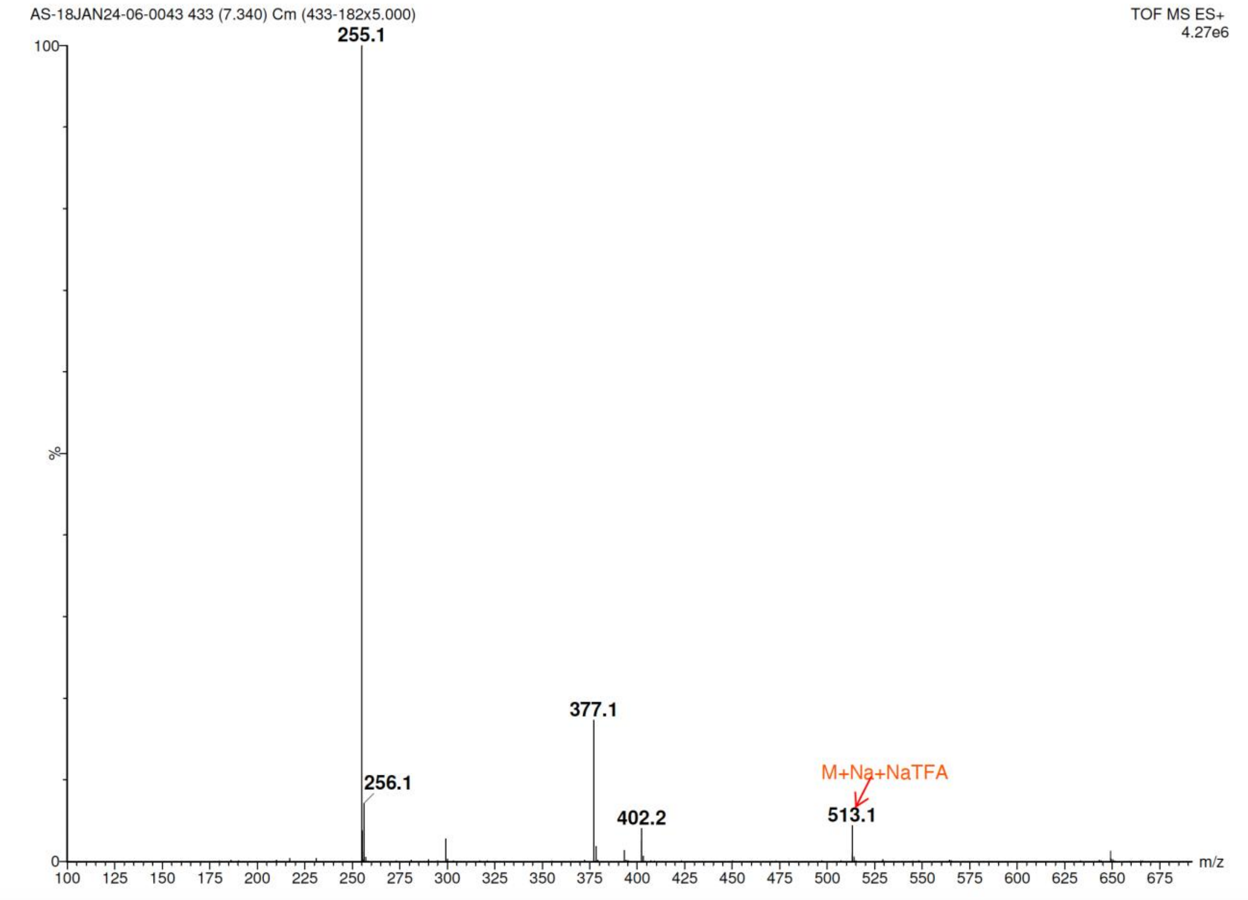

Elemental Composition Report

Page 1

Single Mass Analysis

Tolerance = 5.0 mDa / DBE: min = -1.5, max = 100.0
Element prediction: Off
Number of isotope peaks used for i-FIT = 3

Monoisotopic Mass, Even Electron Ions
55 formula(e) evaluated with 1 results within limits (up to 50 closest results for each mass)
Elements Used:
C: 0-200 H: 0-200 N: 4-4 O: 0-30 23Na: 1-1
AS-18JAN24-06-0043 437 (7.408) AM2 (Ar,25000.0,0.00,0.00); ABS
TOF MS ES+

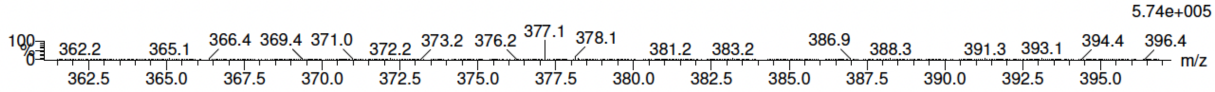

Mass	Calc. Mass	mDa	PPM	DBE	i-FIT	Norm	Conf(%)	Formula
Minimum:				-1.5				
Maximum:		5.0	5.0	100.0				
377.1442	377.1437	0.5	1.3	6.5	489.7	n/a	n/a	C15 H22 N4 O6 23Na

^{1}H and ^{13}C NMR spectra of compound **7**:

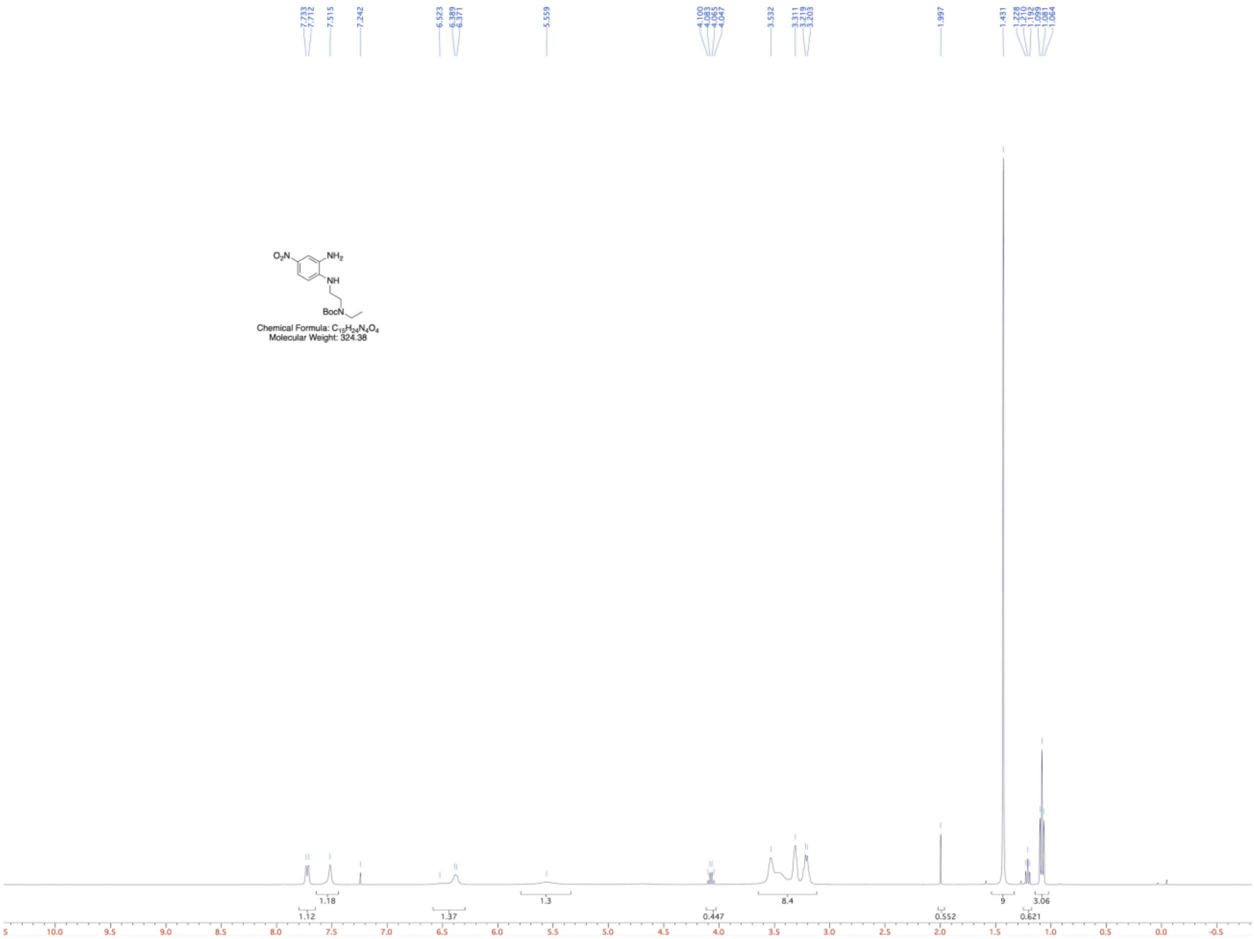

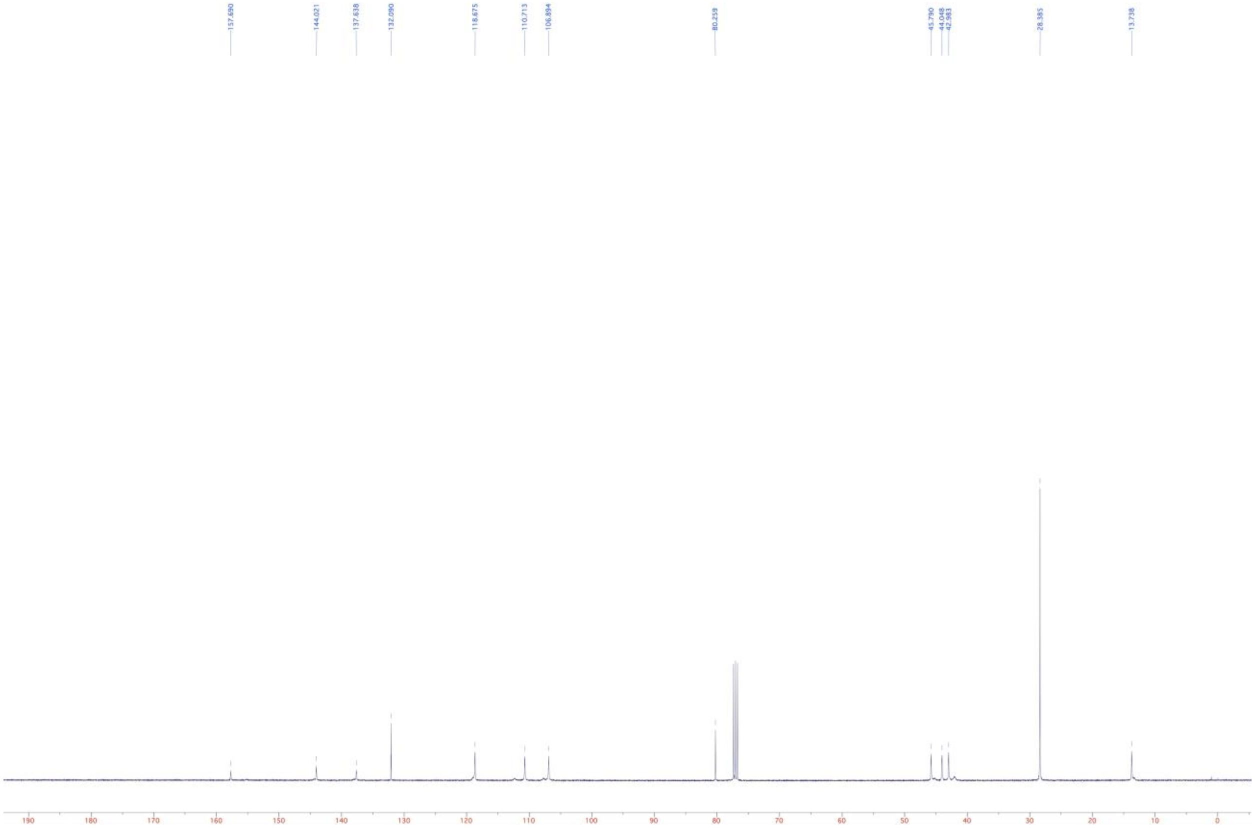

HRMS data of compound **7**:

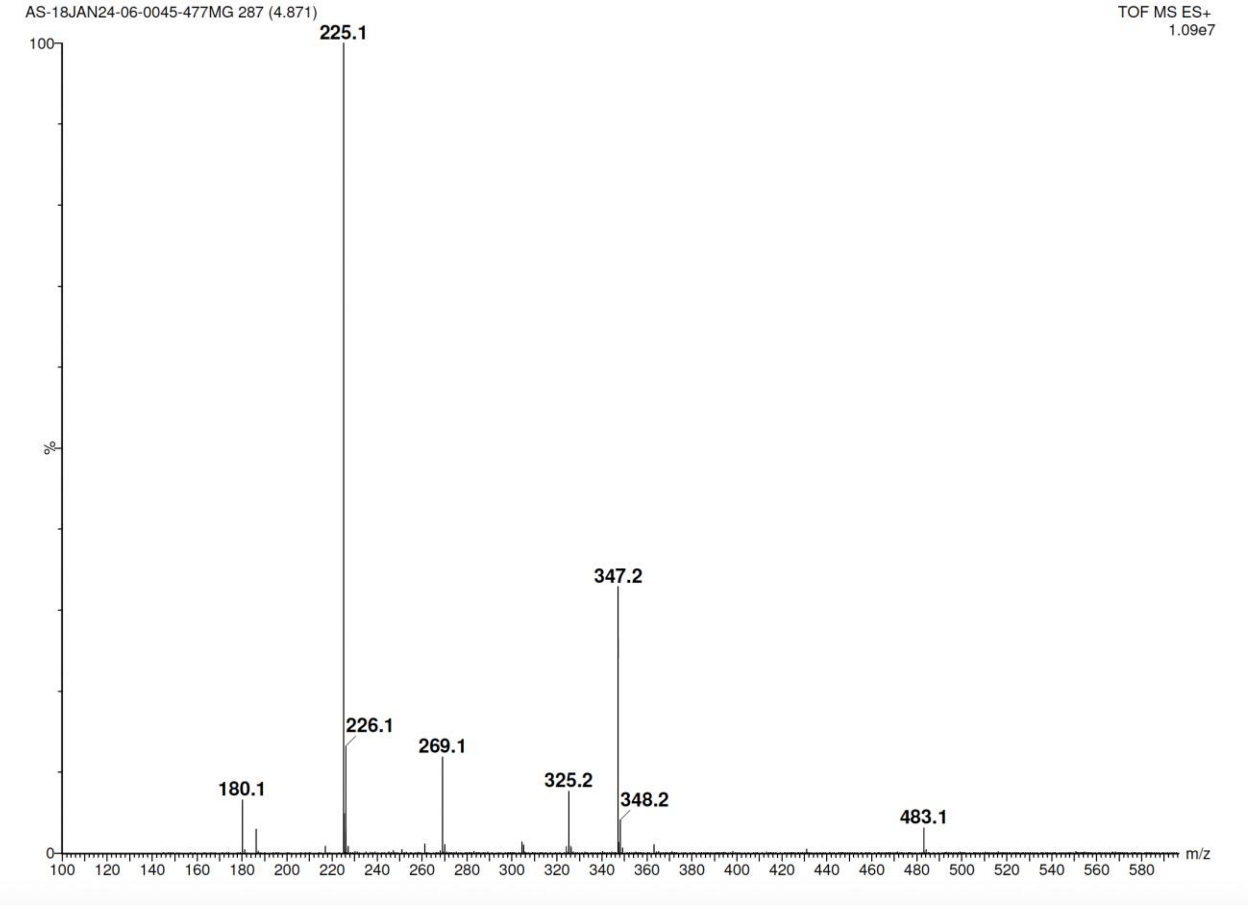

Elemental Composition Report

Page 1

Single Mass Analysis

Tolerance = 5.0 mDa / DBE: min = -1.5, max = 100.0
Element prediction: Off
Number of isotope peaks used for i-FIT = 3

Monoisotopic Mass, Even Electron Ions
50 formula(e) evaluated with 1 results within limits (up to 50 closest results for each mass)
Elements Used:
C: 0-200 H: 0-200 N: 4-4 O: 0-30
AS-18JAN24-06-0045-477MG 291 (4.939) AM2 (Ar,25000.0,0.00,0.00); ABS
TOF MS ES+

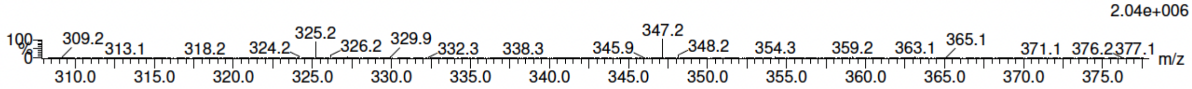

Minimum:				-1.5				
Maximum:		5.0	5.0	100.0				
Mass	Calc. Mass	mDa	PPM	DBE	i-FIT	Norm	Conf(%)	Formula
325.1871	325.1876	-0.5	-1.5	5.5	649.2	n/a	n/a	C15 H25 N4 O4

^{1}H and ^{13}C NMR spectra of compound **8**:

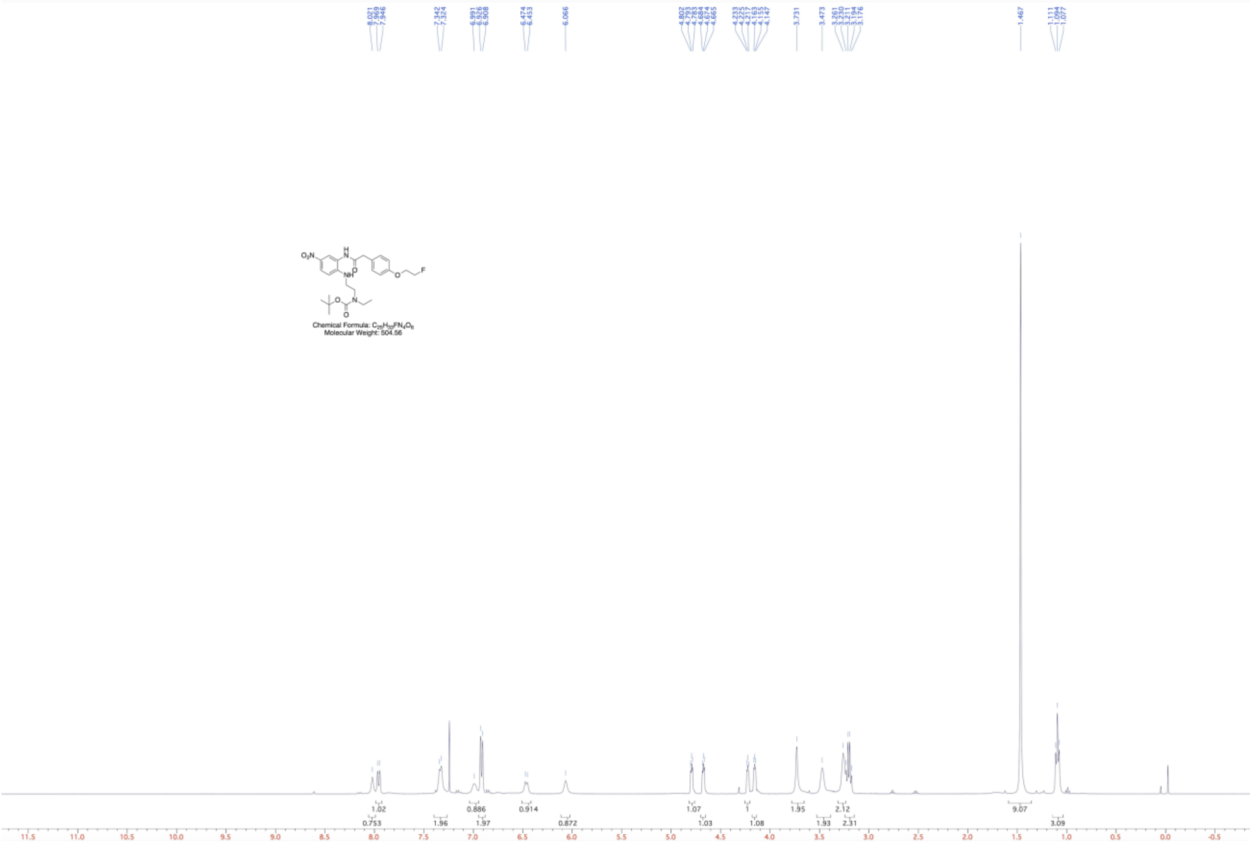

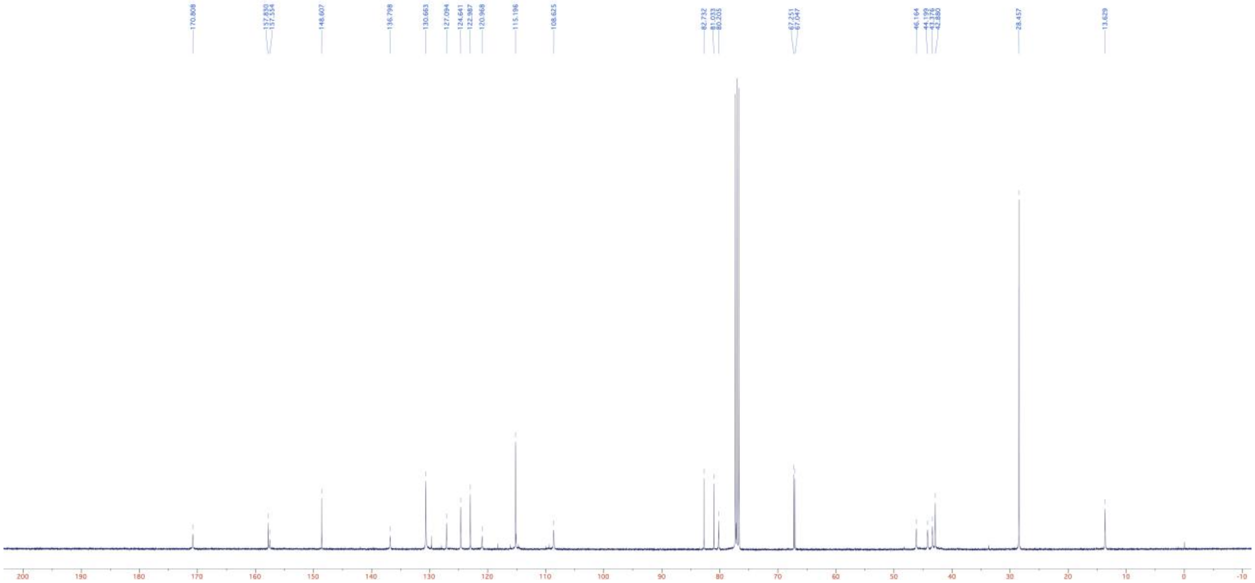

HRMS data of compound **8**:

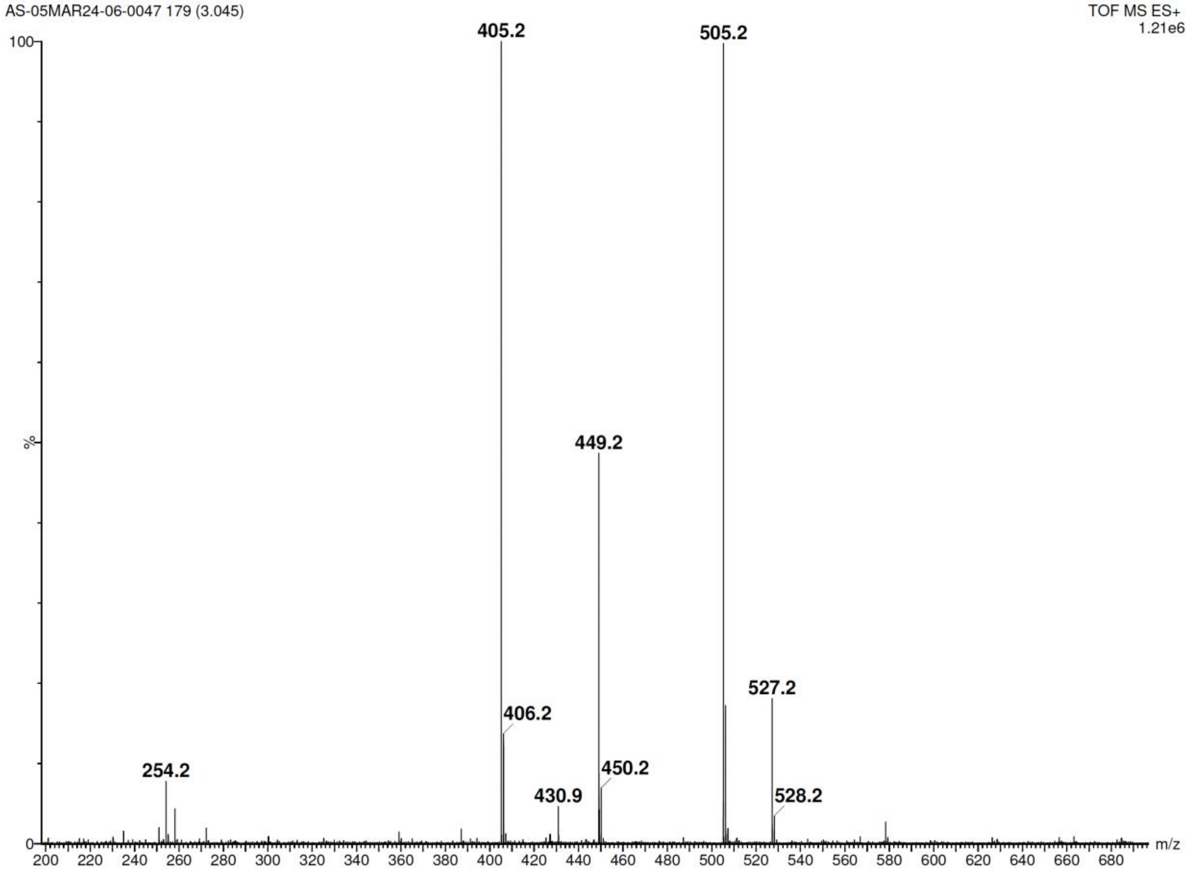

Elemental Composition Report

Page 1

Single Mass Analysis

Tolerance = 5.0 mDa / DBE: min = -1.5, max = 100.0
Element prediction: Off
Number of isotope peaks used for i-FIT = 3

Monoisotopic Mass, Even Electron Ions
100 formula(e) evaluated with 1 results within limits (up to 50 closest results for each mass)
Elements Used:
C: 0-200 H: 0-200 N: 4-4 O: 0-30 F: 1-1
AS-05MAR24-06-0047 183 (3.112) AM2 (Ar,25000.0,0.00,0.00); ABS
TOF MS ES+

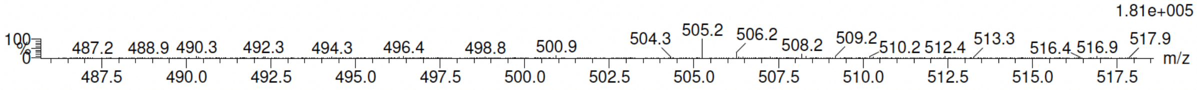

Minimum:				-1.5				
Maximum:		5.0	5.0	100.0				
Mass	Calc. Mass	mDa	PPM	DBE	i-FIT	Norm	Conf(%)	Formula
505.2461	505.2462	-0.1	-0.2	10.5	433.3	n/a	n/a	C25 H34 N4 O6 F

^{1}H and ^{13}C NMR spectra of compound **9**:

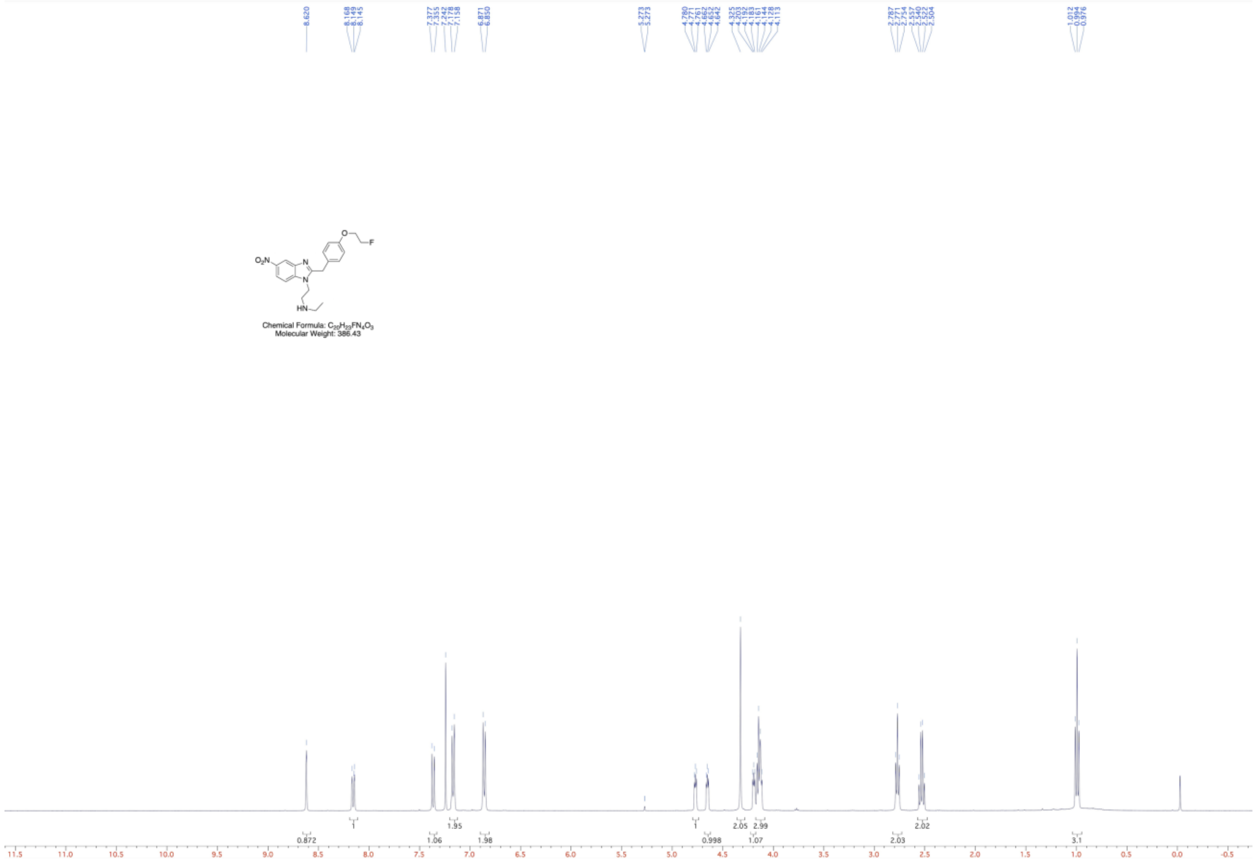

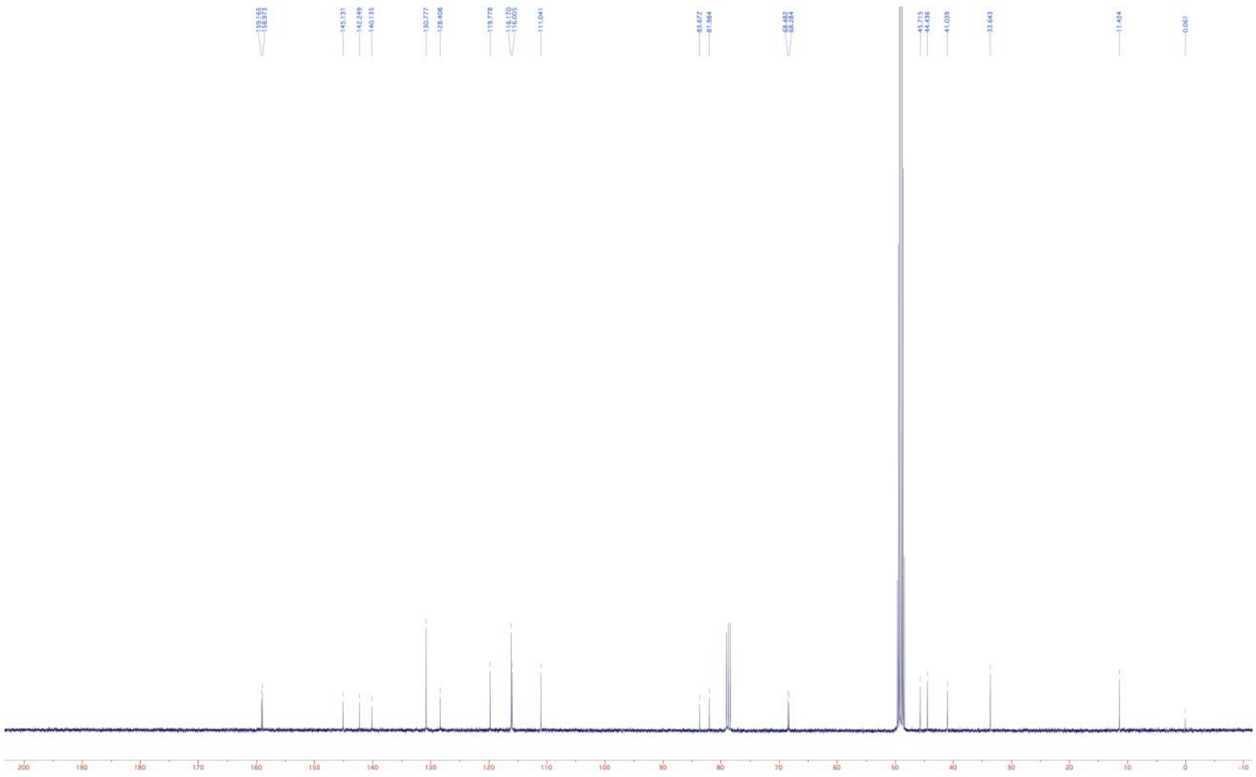

HRMS data of compound **9**:

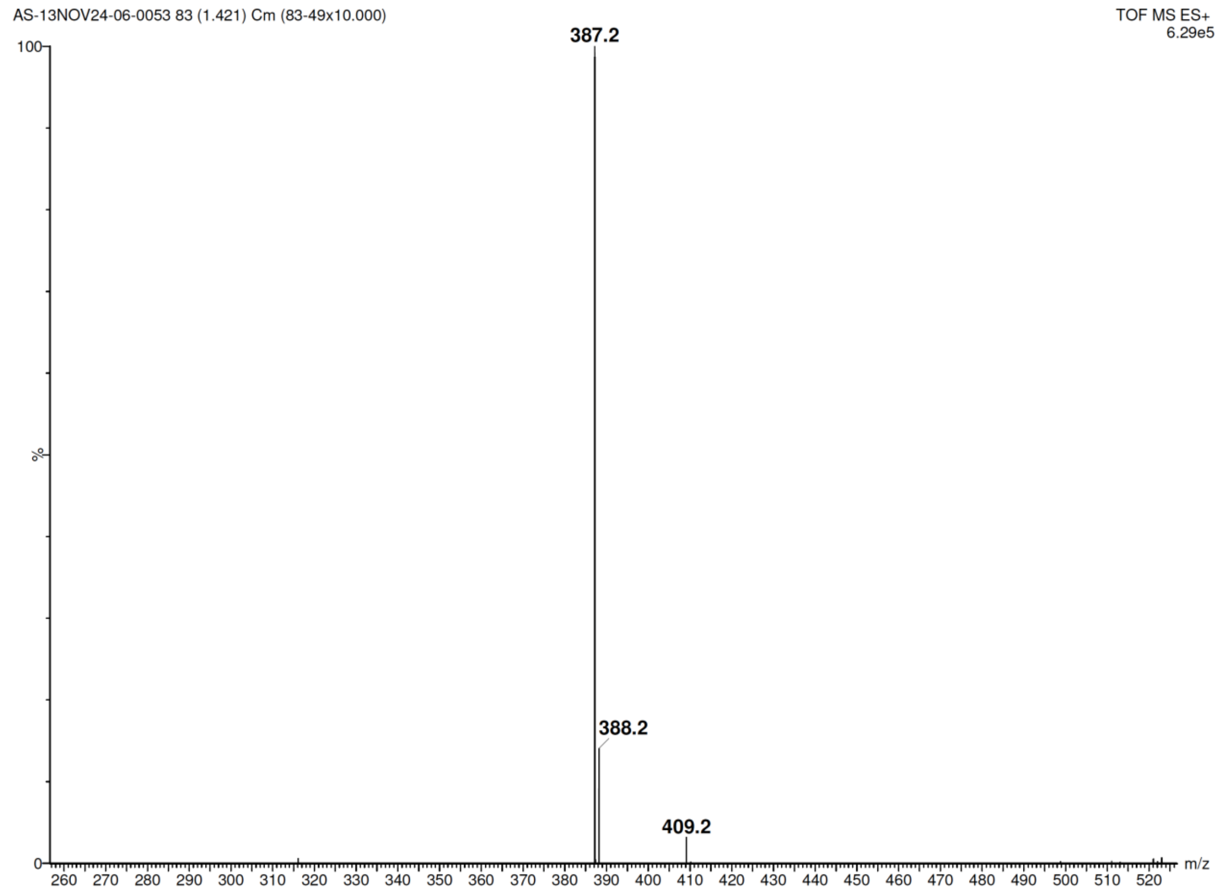

Elemental Composition Report

Page 1

Single Mass Analysis

Tolerance = 5.0 mDa / DBE: min = -1.5, max = 100.0
Element prediction: Off
Number of isotope peaks used for i-FIT = 3

Monoisotopic Mass, Even Electron Ions
63 formula(e) evaluated with 1 results within limits (up to 50 closest results for each mass)
Elements Used:
C: 0-200 H: 0-200 N: 4-4 O: 0-20 F: 1-1
AS-13NOV24-06-0053 87 (1.488) AM2 (Ar,22000.0,0.00,0.00); ABS
TOF MS ES+

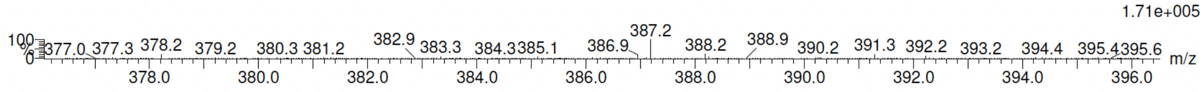

	Calc. Mass	mDa	PPM	DBE	i-FIT	Norm	Conf(%)	Formula
Minimum:				-1.5				
Maximum:		5.0	5.0	100.0				
Mass	Calc. Mass	mDa	PPM	DBE	i-FIT	Norm	Conf(%)	Formula
387.1837	387.1832	0.5	1.3	10.5	454.7	n/a	n/a	C20 H24 N4 O3 F

^{1}H and ^{13}C NMR spectra of compound **10**:

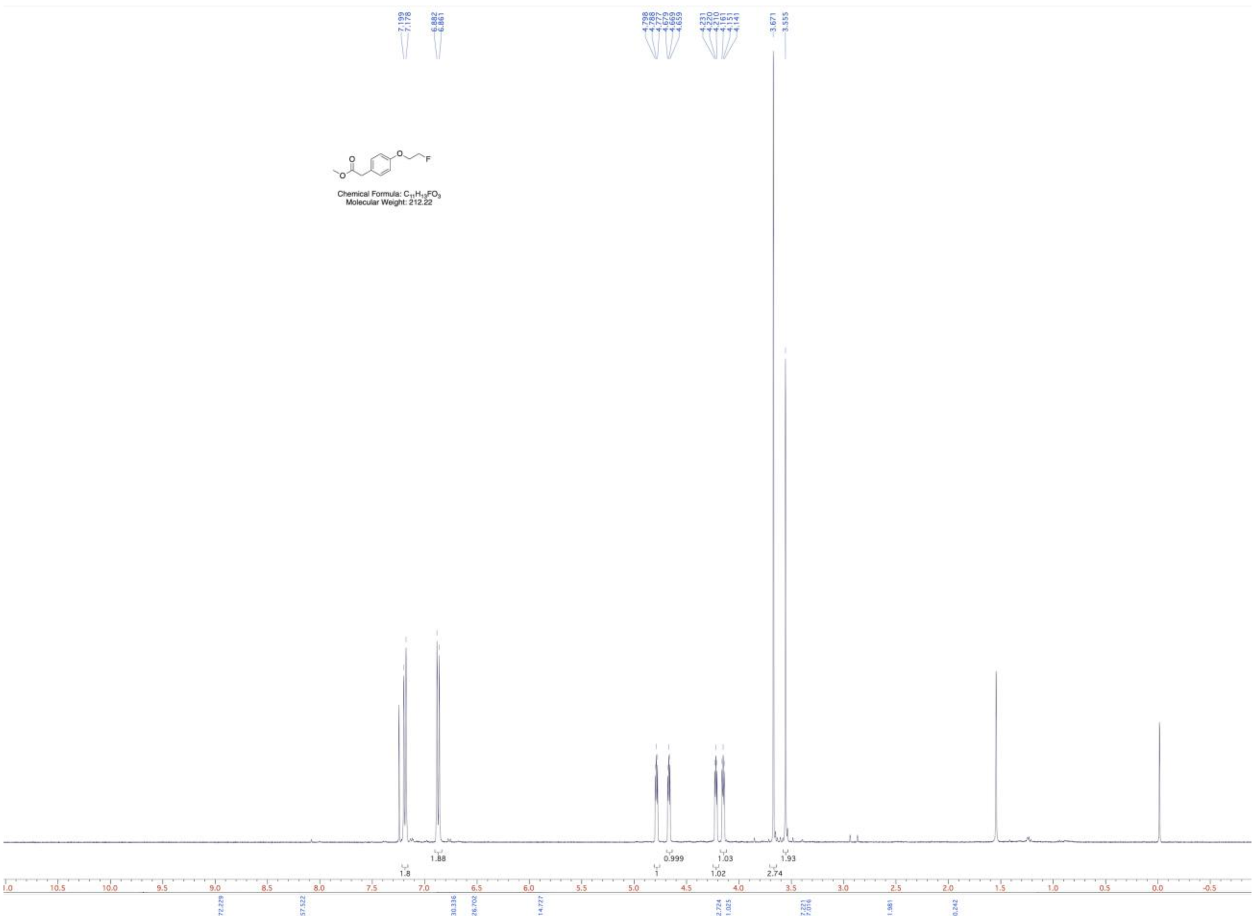

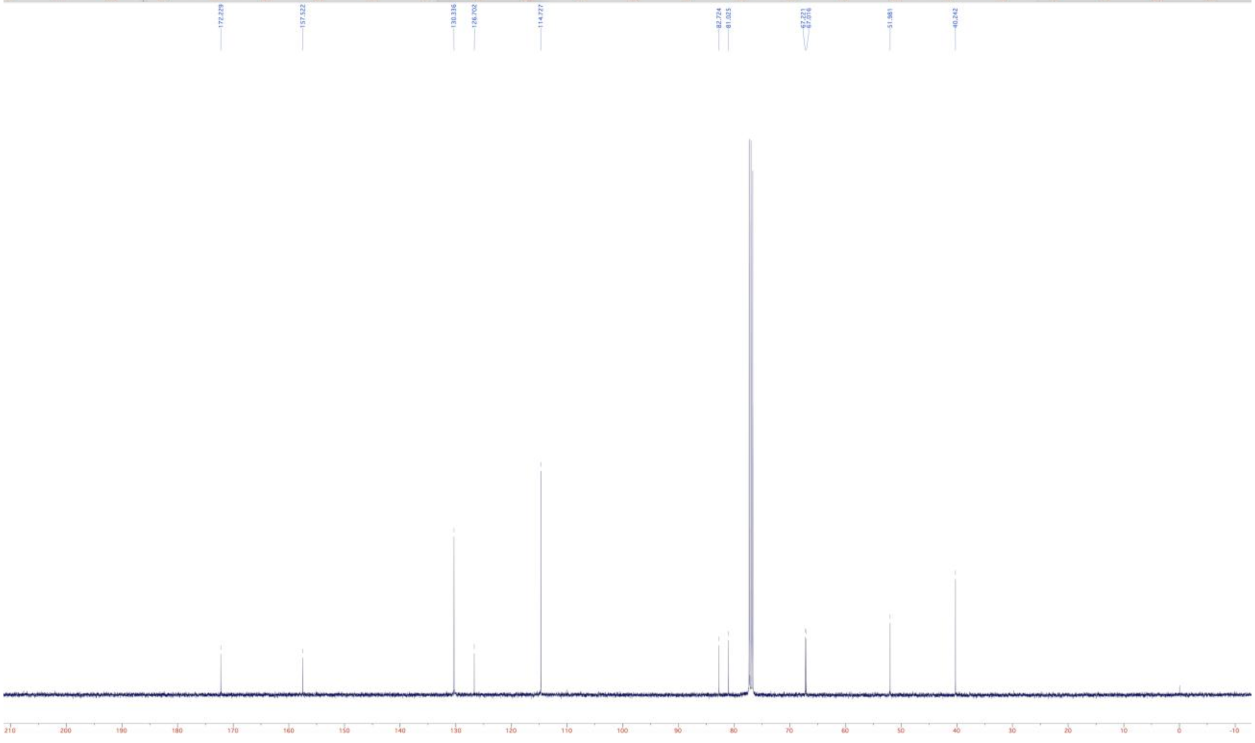

^{1}H and ^{13}C NMR spectra of compound **11**:

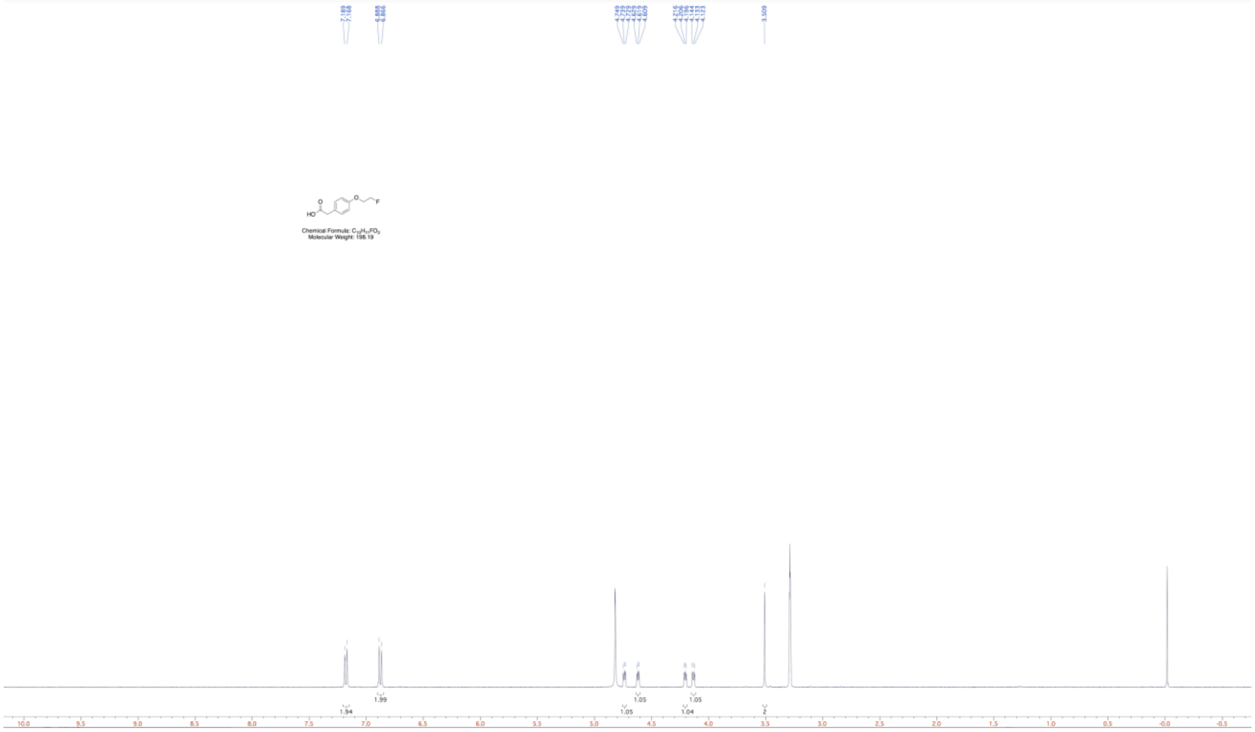

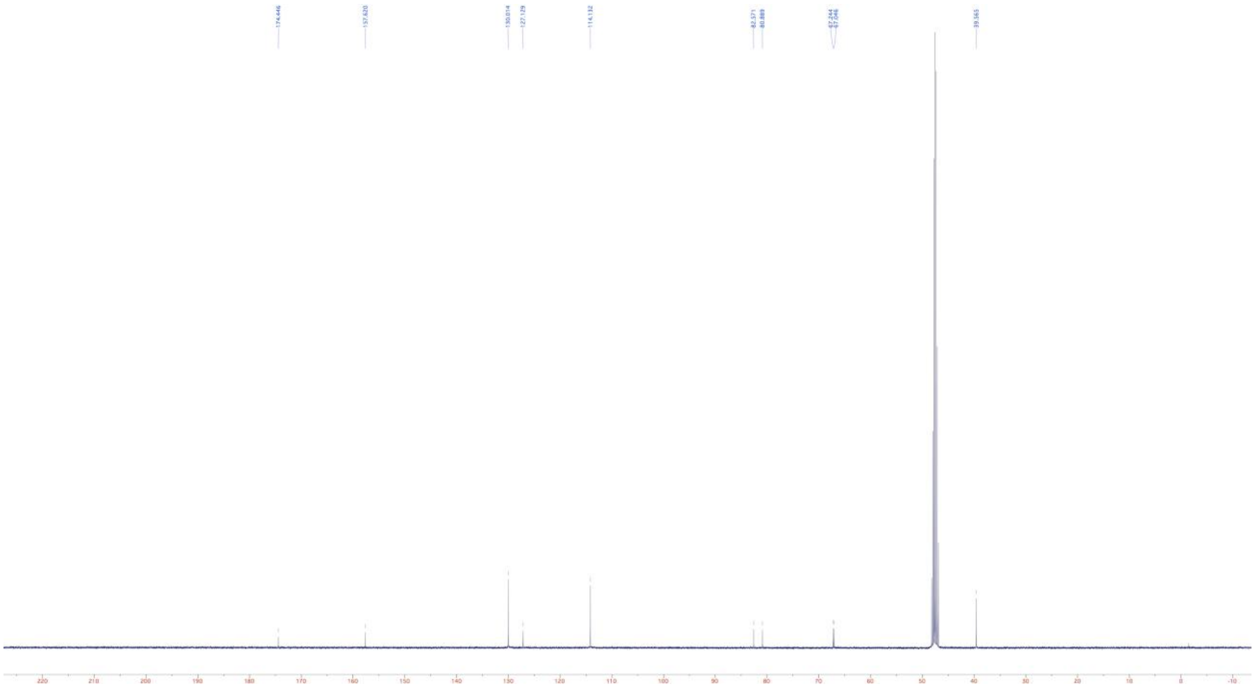

References

1 Hunger, A., Kebrle, J., Rossi, A. & Hoffmann, K. Benzimidazol-Derivate und verwandte Heterocyclen III. Synthese von 1-Aminoalkyl-2-nenzyl-nitro-benzimidazolen. *Helvetica Chimica Acta* **43**, 1032–1046 (1960).

2 Renton, P., Green, B., Maddaford, S., Rakhit, S. & Andrews, J. S. NOpiates: Novel Dual Action Neuronal Nitric Oxide Synthase Inhibitors with µ-Opioid Agonist Activity. *ACS Med Chem Lett* **3**, 227–231 (2012). https://doi.org/10.1021/ml200268w

3 Modemann, D. J. *et al.* Development of high-affinity fluorinated ligands for cannabinoid subtype 2 receptor, and in vitro evaluation of a radioactive tracer for imaging. *Eur J Med Chem* **232**, 114138 (2022). https://doi.org/10.1016/j.ejmech.2022.114138
