# Supplementary material for: A µ-opioid receptor superagonist analgesic with minimal adverse effects
Source: Nature. 2026 Apr 1;652(8112):1393–404. doi: 10.1038/s41586-026-10299-9 (PMC13128446; doi:10.1038/s41586-026-10299-9)
Supplement: Supplementary file 2 — Reporting Summary [file 41586_2026_10299_MOESM2_ESM.pdf]

nature portfolio

Corresponding author(s): Skiniotis, Rice, Michaelides

Last updated by author(s): Jan 30, 2026

Reporting Summary

Nature Portfolio wishes to improve the reproducibility of the work that we publish. This form provides structure for consistency and transparency in reporting. For further information on Nature Portfolio policies, see our Editorial Policies and the Editorial Policy Checklist.

Statistics

For all statistical analyses, confirm that the following items are present in the figure legend, table legend, main text, or Methods section.

n/a	Confirmed	
☐	☒	The exact sample size (*n*) for each experimental group/condition, given as a discrete number and unit of measurement
☐	☒	A statement on whether measurements were taken from distinct samples or whether the same sample was measured repeatedly
☐	☒	The statistical test(s) used AND whether they are one- or two-sided<br>*Only common tests should be described solely by name; describe more complex techniques in the Methods section.*
☐	☒	A description of all covariates tested
☐	☒	A description of any assumptions or corrections, such as tests of normality and adjustment for multiple comparisons
☐	☒	A full description of the statistical parameters including central tendency (e.g. means) or other basic estimates (e.g. regression coefficient) AND variation (e.g. standard deviation) or associated estimates of uncertainty (e.g. confidence intervals)
☐	☒	For null hypothesis testing, the test statistic (e.g. *F*, *t*, *r*) with confidence intervals, effect sizes, degrees of freedom and *P* value noted<br>*Give P values as exact values whenever suitable.*
☒	☐	For Bayesian analysis, information on the choice of priors and Markov chain Monte Carlo settings
☒	☐	For hierarchical and complex designs, identification of the appropriate level for tests and full reporting of outcomes
☒	☐	Estimates of effect sizes (e.g. Cohen's *d*, Pearson's *r*), indicating how they were calculated

Our web collection on statistics for biologists contains articles on many of the points above.

Software and code

Policy information about availability of computer code

Data collection	MED-PC v. 4.2 (Med Associates), Synapse Software v. 95-44132P (Tucker Davis) , Neuroscience Studio v6 (Doric), Nucline NanoScan 3.04.025 (Mediso), Multigauge v3 (Fujifilm), Spectronaut v19.7 (Biognosys), Microbeta2 workstation software (Revvity), Serial EM (ver. 3.9), EPU (v. 2.10).
Data analysis	Microsoft Excel 2024 and 2025, GraphPad Prism 10, MatlabR2016 & MatlabR2023b, Statistical Parametric Mapping (SPM12), PMOD v4.1, Multigauge v3 (Fujifilm), MSStats v.4.10.1, Xcalibur v.4.4.16.14 (ThermoFisher), Neuroscience Studio v6 (Doric). Code for photometry analysis is available at Github (https://github.com/wdunne3/Calcium-Fiber-Photometry-Analysis/ and https://github.com/BonaventuraLab/fiber-photometry). RELION-4.0, cryoSPARC (v4.0), CTFFIND4, and MotionCor2 were used to process cryoEM data. Molecular model was carried out using Coot (version 0.9.8.1 EL) and Phenix (version 1.20.1-4487). GemSpot pipeline utility of Maestro (v.13.8) (Schrodinger) was used to dock ligands.

For manuscripts utilizing custom algorithms or software that are central to the research but not yet described in published literature, software must be made available to editors and reviewers. We strongly encourage code deposition in a community repository (e.g. GitHub). See the Nature Portfolio guidelines for submitting code & software for further information.

Data

Policy information about availability of data

All manuscripts must include a data availability statement. This statement should provide the following information, where applicable:

- Accession codes, unique identifiers, or web links for publicly available datasets
- A description of any restrictions on data availability
- For clinical datasets or third party data, please ensure that the statement adheres to our policy

The authors declare that the data supporting the findings of this study are available within the paper and its Supplementary Information files. The cryoEM density map has been deposited in the Electron Microscopy Data Bank under accession code EMD-70069 (global refinement), EMD-70070 (local refinement, receptor), EMD-70071 (composite map) and the coordinates have been deposited in the Protein Data Bank under accession number 9O36. The mass spectrometry proteomics data have been deposited to the ProteomeXchange Consortium via the PRIDE (https://www.ebi.ac.uk/pride/) partner repository71 with the dataset identifier PXD062863. Reviewers can access these data using the project accession PXD062863 and reviewer token "62GfWQNbF6w3". Alternatively, these data may also be accessed by logging into the PRIDE website using the reviewer username "reviewer_pxd062863@ebi.ac.uk" and password "mmgadjWUcRls". The PDB:6DDE dataset is available at https://www.rcsb.org/structure/6DDE. Source data are provided in the manuscript. Should any other data be needed they are available from the corresponding author upon reasonable request.

Research involving human participants, their data, or biological material

Policy information about studies with human participants or human data. See also policy information about sex, gender (identity/presentation), and sexual orientation and race, ethnicity and racism.

Reporting on sex and gender	N/A
Reporting on race, ethnicity, or other socially relevant groupings	N/A
Population characteristics	N/A
Recruitment	N/A
Ethics oversight	N/A

Note that full information on the approval of the study protocol must also be provided in the manuscript.

Field-specific reporting

Please select the one below that is the best fit for your research. If you are not sure, read the appropriate sections before making your selection.

☒ Life sciences ☐ Behavioural & social sciences ☐ Ecological, evolutionary & environmental sciences

For a reference copy of the document with all sections, see nature.com/documents/nr-reporting-summary-flat.pdf

Life sciences study design

All studies must disclose on these points even when the disclosure is negative.

Sample size	Sample sizes were estimated based on experience from relevant past work in our laboratories and prior literature (e.g. J Neurophysiol. 2026 Jan 1;135(1):130-141. doi: 10.1152/jn.00504.2025, Nature. 2025 Oct;646(8085):746-753. doi: 10.1038/s41586-025-09427-8, Neuropsychopharmacology. 2017 Jun;42(7):1548-1556. doi: 10.1038/npp.2017.4, and Mol Psychiatry. 2024 Mar;29(3):624-632. doi: 10.1038/s41380-023-02353-z.).
Data exclusions	Animals were excluded if they lost catheter patency or due to lack of photometry signal. Rats for self-administration were excluded for sessions where the infusion line disconnected from the rat's catheter port. Photometry data were excluded for sessions where the patchcord became disconnected from the implanted fiber.
Replication	Detailed methods are provided to aid in replication by others. Behavioral (analgesia, locomotor, hypoxia, tolerance, self-administration) and photometry experiments were replicated in at least 2 cohorts or species and by more than one experimenter. Pharmacokinetic experiments were replicated in at least two species. PET studies were replicated across individual animals. In vitro assays (binding and functional) were replicated in at least two different laboratories and were performed with at least two independent experiments. All other in vitro assays were replicated with at least two independent replicates. All replication attempts were successful.
Randomization	Animals were randomly assigned to experimental groups and treatment conditions. Samples for in vitro assays were randomized with respect to treatment conditions.
Blinding	Experimenters were not blinded to group allocation during data collection for most in vivo experiments because data were collected in an

Blinding	automated manner without manual scoring. Data were analyzed blind if applicable (e.g., PET, photometry) because of automated analysis but experimenters were always aware of the conditions. Withdrawal experiments were performed blinded as noted in the manuscript.

Reporting for specific materials, systems and methods

We require information from authors about some types of materials, experimental systems and methods used in many studies. Here, indicate whether each material, system or method listed is relevant to your study. If you are not sure if a list item applies to your research, read the appropriate section before selecting a response.

Materials & experimental systems

n/a	Involved in the study	
☒	☐	Antibodies
☐	☒	Eukaryotic cell lines
☒	☐	Palaeontology and archaeology
☐	☒	Animals and other organisms
☒	☐	Clinical data
☒	☐	Dual use research of concern
☒	☐	Plants

Methods

n/a	Involved in the study	
☒	☐	ChIP-seq
☒	☐	Flow cytometry
☒	☐	MRI-based neuroimaging

Eukaryotic cell lines

Policy information about cell lines and Sex and Gender in Research

Cell line source(s)	ATCC CRL-321, HEK-293, HEK293T
Authentication	Cell lines were not authenticated after receipt from ATCC
Mycoplasma contamination	Cell lines were tested for mycoplasma contamination as noted in the manuscript. If contaminated cultures were detected, they were not used for experiments.
Commonly misidentified lines (See ICLAC register)	HEK cells can be misidentified as HeLa cells but HeLa cells were not used in these experiments.

Animals and other research organisms

Policy information about studies involving animals; ARRIVE guidelines recommended for reporting animal research, and Sex and Gender in Research

Laboratory animals	Sprague-Dawley rats (Charles River) (6-8 weeks old), C57BL/6J mice (Charles River or Jackson Laboratory) (8 weeks old), TH-cre mice (B6.Cg-7630403G23RikTg(Th-cre)1Tmd/J, NIDA) (10-12 weeks old), Oprm1 knockout mice (B6.129S2-Oprm1tm1Kff/J, Jackson Laboratory) (6-8 weeks old), P-gp/Bcrp knockout mice (FVB.129P2-Abcb1atm1BorAbcb1btm1BorAbcg2tm1Ahs, Taconic Biosciences) (6-8 weeks old).
Wild animals	The study did not involve wild animals
Reporting on sex	We used male and female rats and mice for all experiments unless specified in the methods.
Field-collected samples	The study did not involve animals collected from the field
Ethics oversight	Experiments and procedures complied with ethical regulations for animal testing and research, followed the NIH guidelines and were approved by the relevant institutional animal care and use committees (NIDA, Boston University, University of Barcelona).

Note that full information on the approval of the study protocol must also be provided in the manuscript.

Plants

Seed stocks	*Report on the source of all seed stocks or other plant material used. If applicable, state the seed stock centre and catalogue number. If plant specimens were collected from the field, describe the collection location, date and sampling procedures.*
Novel plant genotypes	*Describe the methods by which all novel plant genotypes were produced. This includes those generated by transgenic approaches, gene editing, chemical/radiation-based mutagenesis and hybridization. For transgenic lines, describe the transformation method, the number of independent lines analyzed and the generation upon which experiments were performed. For gene-edited lines, describe the editor used, the endogenous sequence targeted for editing, the targeting guide RNA sequence (if applicable) and how the editor was applied.*
Authentication	*Describe any authentication procedures for each seed stock used or novel genotype generated. Describe any experiments used to assess the effect of a mutation and, where applicable, how potential secondary effects (e.g. second site T-DNA insertions, mosiacism, off-target gene editing) were examined.*
